# Ventilatory ratio as a predictor for extubation failure in critical ill patients based on MIMIC-IV database (from 2008 to 2019)

**DOI:** 10.3389/fphys.2023.1137115

**Published:** 2023-06-01

**Authors:** Huan Yang, Yuenan Ni, Dong Huang, Zongan Liang

**Affiliations:** Department of Respiratory and Critical Care Medicine, West China School of Medicine and West China Hospital, Sichuan University, Chengdu, China

**Keywords:** ventilatory ratio, extubation failure, mechanical ventilation, intensive care unit, prediction

## Abstract

**Background:** The predictive ability of the ventilatory ratio (VR) for extubation failure risk in critically ill patients on mechanical ventilation is unclear. This study aims to examine the predictive ability of VR for extubation failure risk.

**Methods:** This retrospective study was based on the MIMIC-IV database. The MIMIC-IV database consists of the clinical information of patients who were admitted to the intensive care unit at the Beth Israel Deaconess Medical Center between 2008 and 2019. With extubation failure as the primary outcome and in-hospital mortality as the secondary outcome, we assessed the predictive value of VR 4 hours before extubation using a multivariate logistic regression model.

**Results:** Of 3,569 ventilated patients who were included, the rate of extubation-failure was 12.7% and the median Sequential Organ Failure Assessment (SOFA) score was 6 before extubation. Increased VR, elevated heart rate, greater positive end-expiratory pressure, higher blood urea nitrogen level, higher platelet count, greater SOFA score, decreased pH, decreased tidal volume, presence of chronic pulmonary disease, paraplegia, and metastatic solid tumor were independent predictors for extubation failure. A threshold of 1.595 of VR was associated with prolonged intensive care unit length of stay, higher risk of mortality and extubation failure. The area under the receiver operating characteristic curve (ROC) for VR was 0.669 [0.635–0.703], which was significantly larger than the rapid shallow breathing index [0.510 (0.476–0.545)] and the partial pressure of oxygen to the fraction of inspired oxygen [0.586 (0.551–0.621)].

**Conclusion:** VR 4 hours before extubation was associated with extubation failure, mortality, and prolonged length of stay in the intensive care unit. VR provides good predictive performance for extubation failure (measured by ROC) than the rapid shallow breathing index. Further prospective studies are warranted to confirm these findings.

## Background

Invasive mechanical ventilation (IMV) is an advanced respiratory support widely used in intensive care units (ICUs). The process of removing an endotracheal tube to liberate a patient from mechanical ventilation (MV) is referred to as extubation. Previous studies have found that reintubation after extubation failure was associated with higher morbidity and mortality (Boles et al., 2007; Thille et al., 2013). To avoid prematurely failed extubation attempts while avoiding unnecessary prolonged MV days, many factors such as rapid shallow breathing index (RSBI), maximum occlusion pressure, etc., were assessed by prior studies (Frutos-Vivar et al., 2006; Eskandar and Apostolakos, 2007; Hsieh et al., 2018). However, no ideal parameters were proven to have a high predictive value for extubation. Moreover, those studies focused on oxygenation-related indices other than ventilation-related indices in most cases, while the ability to eliminate carbon dioxide (CO_2_) was found to be associated with disease progression and prognosis (Nuckton et al., 2002; Cepkova et al., 2007; Siddiki et al., 2010). Therefore, it is meaningful to accurately predict the risk of extubation failure and add additional ventilation-related information to facilitate the extubation process.

The ventilatory ratio (VR) is a simple bedside index of ventilation efficiency (Sinha et al., 2009). VR is defined as the ratio of the observed minute volume and observed partial pressure of arterial carbon dioxide (P_a_CO_2_) over the predicted minute volume and predicted P_a_CO_2_. The predicted minute volume is calculated with the predicted body weight, and a value that lies close to the mean P_a_CO_2_ in healthy individuals represents the predicted P_a_CO_2_. A value of VR approximating one represents matched perfusion ventilation that can clear CO_2_ efficiently (Sinha et al., 2009). Several studies reported that higher VR was associated with higher mortality and shorter ventilator-free days in patients with acute respiratory distress syndrome (ARDS), reflecting the severity of the disease (Sinha et al., 2013a; Sinha et al., 2013b; Sinha et al., 2014; Sinha et al., 2019; Ende et al., 2021; Torres et al., 2021). Previous studies also showed the potential value of VR to adjust strategies to liberate from ventilators. One study found that patients were finally liberated from ventilators only when VR fell under 2 and higher VR was related to a prolonged duration of the weaning process (Proklou et al., 2021). However, to our knowledge, there is no study discussing the predictive ability of VR obtained before extubation for the risk of extubation failure. This study aims to identify the association between VR and extubation failure and explore the cut-off value of VR as a tool to facilitate the decision-making process of extubation in clinical situations.

## Methods

### Source of data

This study was a retrospective cohort analysis using the open-source critical care database MIMIC-IV (Version 1.0) (Goldberger et al., 2000; Johnson et al., 2011). The MIMIC-IV database consists of the clinical information of patients who were admitted to the ICU at the Beth Israel Deaconess Medical Center between 2008 and 2019. One author, Huan Yang, completed the Collaborative Institutional Training Initiative examination (certification number: 45308698) to achieve access to the database for data extraction.

### Outcome definition and data collection

Extubation failure was defined as the need for ventilatory support [noninvasive ventilation (NIV) or reintubation] or death within 48 h following extubation (Boles et al., 2007). We extracted variables with PostgreSQL (Version 14.0; PostgreSQL Global Development Group) and pgAdmin 4 (Version 6.11). The inclusion criteria were as follows: i) age > 18 years, ii) height > 152.4 cm, iii) with extubation records. The exclusion criteria were as follows: i) not the first time of extubation during a hospital stay, ii) unplanned extubation, iii) ICU length of stay (LOS) ≤ 48 h, iv) main data missed: MV settings and P_a_CO_2_ 4 hours before extubation.

Respiratory parameters, arterial blood gas (ABG), and vital signs were extracted 4 hours before extubation. To reduce missing data, other clinical and laboratory variables were extracted within 24 h before extubation, including Glasgow Coma Scales (GCS), the Sequential Organ Failure Assessment (SOFA), blood routines, liver and kidney functions, coagulations, cardiac markers, etc. Reasonable ranges were set for filtering out extreme values (Supplementary Table S10). Observed values outside these ranges were treated as missing data. For some variables with multiple measurements, the worst values were assessed based on clinical relevance. In addition, features (e.g., age, sex, comorbidities) were selected within 24 h after ICU admission. Finally, NIV use, reintubation, and death within 48 h after extubation, in-hospital mortality, ICU LOS, and hospital LOS were also assessed.

Predicted body weight was calculated using the NHLBI ARDS network formula (Acute Respiratory Distress Syndrome Network et al., 2000).

$$female=45.5+0.91*\left(height\;in\;cm-152.4\right)$$

$$male=50+0.91*\left(height\;in\;cm-152.4\right)$$

VR was calculated as follows (Sinha et al., 2009):

$$VR=minute\;volume\;\left[mL\right]\times PaCO2\;\left[mmHg\right]/\left(predicted\;body\;weight\;\left[kg\right]/100\;\left[mL/kg\right]\times 37.5\;\left[mmHg\right]\right)$$

### Statistical analysis

Continuous variables are presented as medians with their interquartile ranges (IQRs), and categorical variables are presented as total numbers and percentages. Normal distribution for continuous variables was determined using the Shapiro‒Wilk test (Razali and Wah, 2011). Continuous variables (median) were compared using Student’s test (normal distribution) (Mishra et al., 2019) or the Wilcoxon rank sum test (nonnormal distribution) (McKnight et al., 2010), and proportions were compared using χ2 or Fisher exact tests when the numbers were small (Hess and Hess, 2017). Statistical significance was considered to be at two-sided *p* < 0.05. The MIMIC-IV dataset was first randomly split into the training set (70%) and the internal validation set (30%). In the training set, a receiver operating characteristic (ROC) curve analysis was used to confirm the best cut-off values of VR, RSBI, and the partial pressure of oxygen to the fraction of inspired oxygen (P_a_O_2_/F_i_O_2_) at 4 h before extubation. The predictive ability of cut-off values was assessed for discrimination using C-statistics in the validation set. Variables with a two-sided *p*-value of < 0.05 from the independent Student’s t-test/Wilcoxon rank sum test and χ2/Fisher exact tests were considered for multivariate logistic regression. Lasso regression was performed for automatic feature selection. Multicollinearity was calculated through the analysis of the variance inflation factors. Thereafter, factors with clinical plausibility were selected and further analyzed in stepwise multiple logistic regression to identify the association between extubation failure and VR. To test the robustness of any association between extubation failure and VR, interactions with other covariates [i.e., age, RSBI, P_a_O_2_/F_i_O_2_, sex, positive end-expiratory pressure (PEEP), SOFA] that increased extubation failure were tested (Thille et al., 2013). Furthermore, a sensitivity analysis was conducted by excluding patients who were admitted for surgical reasons. The results were summarized with the odds ratio (OR), 95% confidence interval (CI), and *p*-value. To avoid bias introduced by missing data, variables with more than 10% missing data were excluded, and the analysis of the primary outcome was imputed using multiple imputation chain equations using the “MICE” package in R. All statistical analyses were performed with R version 4.0.5 (http://www.R-project.org) and Stata/SE 15.1.

## Results

### Study population

**FIGURE 1:**
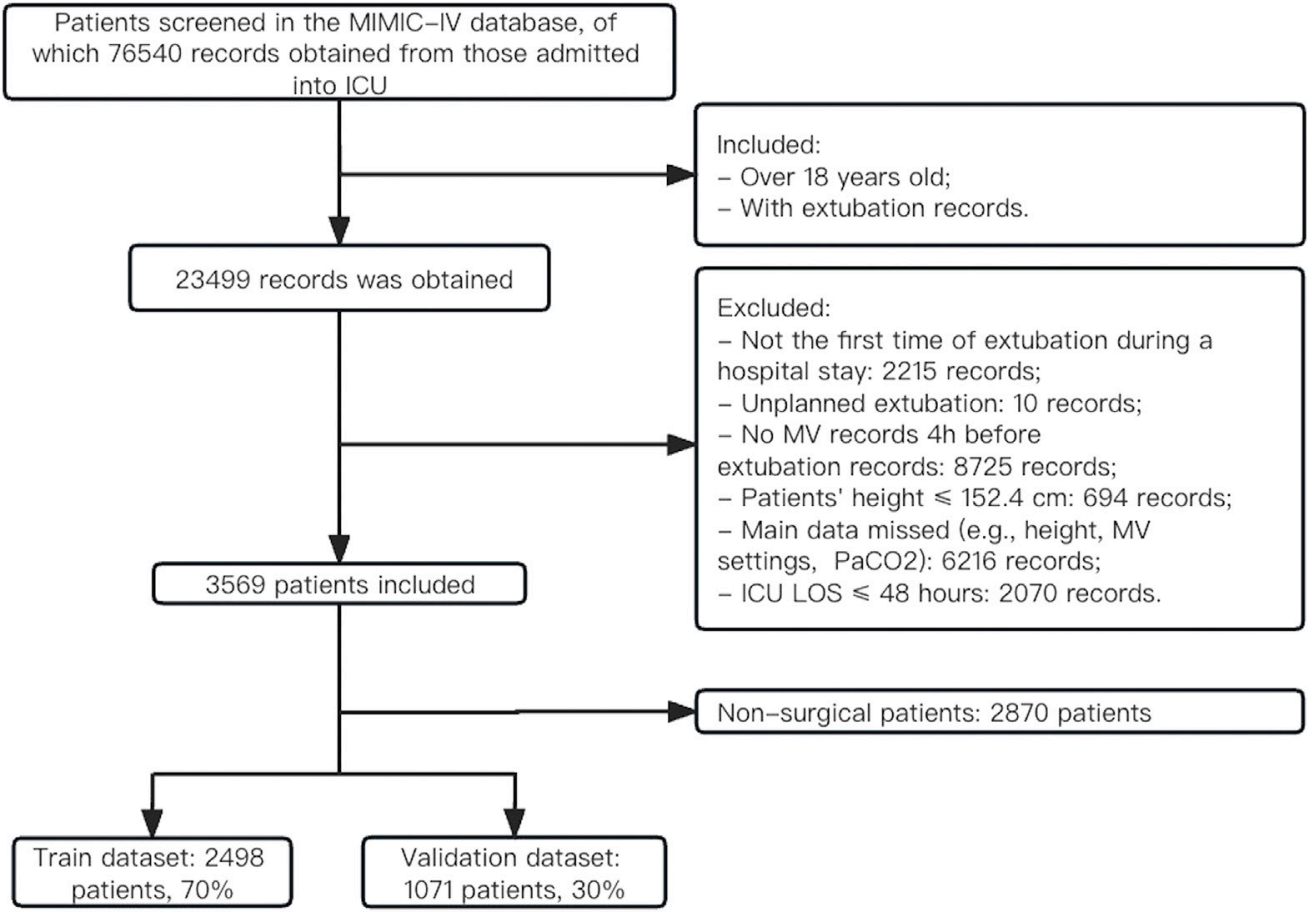
Flowchart of patient selection.

As shown in Figure 1, a total of 76,540 records were obtained from patients who were admitted to the ICU. Of those, 23,499 records were extracted from patients (over 18 years old) who had extubation records. 3,569 patients who underwent planned extubation were ultimately extracted from the MIMIC-IV database according to prespecified inclusion criteria. Overall, the majority of patients (67.1%) were male, and the mean age was 65.29 ± 13.98 years (Supplementary Table S1). The Charlson comorbidity index was 6 (IQR, 4–7) and the median SOFA score was 6 (IQR, 5–9) before extubation. The detailed clinical information for the included patients before extubation is listed in Supplementary Table S1, and the baseline characteristics on ICU admission are presented in Supplementary Table S2. 1839 (56.0%, *n* = 3,284) patients had VR > 2 on admission, and 496 patients (13.9%, *n* = 3,569) had VR > 2 before extubation. The median duration of ICU LOS was 4 days (IQR, 3–7) and that of hospital LOS was 10 days (IQR, 7–16). There were 454 (12.7%) patients who failed extubation within 48 h, and the overall in-hospital mortality was 7.3%. The extracted dataset was randomly divided into the training set (*n* = 2,498) and the internal validation set (*n* = 1,071). The baseline characteristics were similar between the training set and validation set (Supplementary Table S3). For sensitivity analysis, 2,870 non-surgical patients were obtained. The rate of extubation failure was 13.6% (391) and the rate of mortality was 8.7% (250) in this population (see Supplementary Table S4).

### VR and other risk factors

In the training set, patients in the extubation failure group had significantly higher VRs (1.71 [1.36, 2.10] vs. 1.41 [1.19, 1.71], *p* < 0.001). Moreover, the extubation failure group exhibited a greater prevalence of comorbidities, required higher ventilation settings, had higher mean values for heart rate, GCS, SOFA, P_a_CO_2_, anion gap, and a lower mean value of pH, P_a_O_2_/F_i_O_2_, and peripheral arterial oxygen saturation (S_p_O_2_). Notable differences in laboratory variables included higher blood urea nitrogen level (BUN), creatinine level, and platelet count among the extubation failure group (all *p* < 0.05), as shown in Table 1.

**TABLE 1:** Baseline population characteristics with stratification by extubation failure before extubation in the train set.

Variables	Overall	Extubation success	Extubation failure	*p*-value
	2,498	2,192	306	
Age (year)	67.00 [57.00, 76.00]	67.00 [57.00, 76.00]	66.00 [56.00, 74.00]	0.414
Male (*n*, %)	1,687 (67.5)	1,476 (67.3)	211 (69.0)	0.616
BMI (kg/m2)	27.68 [24.19, 31.88]	27.47 [24.13, 31.56]	29.17 [24.80, 34.40]	<0.001
Type of admission (*n*, %)				<0.001
Emergency	846 (33.9)	714 (32.6)	132 (43.1)	
Surgery	491 (19.7)	445 (20.3)	46 (15.0)	
Urgent	640 (25.6)	571 (26.0)	69 (22.5)	
Elective	251 (10.0)	232 (10.6)	19 (6.2)	
Other	270 (10.8)	230 (10.5)	40 (13.1)	
Charlson comorbidity index (score)	6.00 [4.00, 8.00]	6.00 [4.00, 7.00]	6.00 [5.00, 8.00]	<0.001
Chronic pulmonary disease (*n*, %)	712 (28.5)	598 (27.3)	114 (37.3)	<0.001
Paraplegia (*n*, %)	67 (2.7)	50 (2.3)	17 (5.6)	0.002
Metastatic solid tumor (*n*, %)	59 (2.4)	44 (2.0)	15 (4.9)	0.003
Diabetes (*n*, %)	858 (34.3)	737 (33.6)	121 (39.5)	0.048
Liver disease (*n*, %)	274 (11.0)	223 (10.2)	51 (16.7)	0.001
ARDS (*n*, %)	24 (1.0)	17 (0.8)	7 (2.3)	0.026
Heart rate (/min)	88.00 [80.00, 100.00]	88.00 [80.00, 99.00]	92.50 [84.00, 104.75]	<0.001
Glasgow coma scale (score)	15.00 [15.00, 15.00]	15.00 [15.00, 15.00]	15.00 [14.00, 15.00]	0.047
SOFA (score)	6.00 [5.00, 9.00]	6.00 [5.00, 8.00]	7.50 [5.00, 10.00]	<0.001
f (/min)	19.00 [16.00, 24.00]	19.00 [16.00, 24.00]	21.00 [17.00, 26.75]	<0.001
Tidal volume (mL)	496.00 [421.00, 588.75]	500.00 [424.00, 594.00]	479.50 [400.25, 556.75]	0.002
PEEP (cmH2O)	5.00 [5.00, 5.00]	5.00 [5.00, 5.00]	5.00 [5.00, 8.00]	<0.001
RSBI (/[min`mL])	42.02 [30.83, 57.63]	41.97 [30.82, 57.60]	43.49 [31.11, 58.70]	0.564
Ventilatory ratio	1.44 [1.21, 1.75]	1.41 [1.19, 1.71]	1.71 [1.36, 2.10]	<0.001
PaO2/FiO2 (mmHg)	244.00 [184.00, 314.00]	247.50 [187.50, 317.50]	222.00 [159.25, 284.38]	<0.001
SpO2 (%)	95.00 [93.00, 97.00]	95.00 [93.00, 97.00]	94.00 [92.00, 96.00]	<0.001
PaCO2 (mmHg)	40.00 [36.00, 45.00]	40.00 [36.00, 45.00]	42.00 [36.00, 48.00]	0.006
pH	7.38 [7.34, 7.42]	7.38 [7.34, 7.42]	7.37 [7.33, 7.42]	0.005
Anion gap (mEq/L)	13.00 [11.00, 16.00]	13.00 [11.00, 16.00]	14.00 [12.00, 18.00]	<0.001
Blood urea nitrogen (mg/dL)	19.00 [14.00, 30.00]	19.00 [14.00, 28.00]	23.00 [16.00, 41.00]	<0.001
Creatinine (mg/dL)	1.00 [0.80, 1.50]	1.00 [0.80, 1.40]	1.20 [0.80, 2.00]	<0.001
Platelet count (10^9^/L)	138.00 [102.00, 186.00]	137.00 [102.00, 184.00]	150.50 [98.50, 216.75]	0.017
ICU LOS (day)	4 [3, 7]	4 [3, 7]	8 [4, 15]	<0.001
Hospital LOS (day)	10 [7, 16]	10 [7, 15]	14 [9, 23]	<0.001
In-hospital death (*n*, %)	187 (7.5)	96 (4.4)	91 (29.7)	<0.001
Extubation failure type (*n*, %)	306 (12.2)	0 (0.0)	306 (100.0)	<0.001
NIV after extubation (*n*, %)	97 (3.9)	0 (0.0)	97 (31.7)	<0.001
Death within 48 h (*n*, %)	57 (2.3)	0 (0.0)	57 (18.6)	<0.001
Reintubation within 48 h (*n*, %)	159 (6.4)	0 (0.0)	159 (52.0)	<0.001

Definition of abbreviations: BMI body mass index; SOFA the Sequential Organ Failure Assessment; PEEP positive end-expiratory pressure; RSBI rapid shallow breath index; F_i_O_2_ fraction inspired oxygen concentration; P_a_CO_2_ arterial carbon dioxide partial pressure; S_p_O_2_ peripheral arterial oxygen saturation; P_a_O_2_ arterial oxygen partial pressure; ICU intensive care unit; LOS length of stay; NIV noninvasive ventilation. Full table see Supplementary Table 11.

Increased VR was an independent risk factor for extubation failure after multivariable adjustment (OR 2.14 [1.65–2.72], *p* < 0.001). Other factors, including elevated heart rate (OR 1.01 [1.00–1.02]), lower tidal volume (OR 1.00 [1.00–1.00]), higher PEEP (OR 1.08 [1.01–1.15]), increased BUN (OR 1.01 [1.01–1.02]), increased platelet count (OR 1.00 [1.00–1.00]), greater SOFA (OR 1.12 [1.07–1.17]), presence of chronic pulmonary disease (OR 1.33 [1.01–1.74]), presence of paraplegia (OR 2.44 [1.29–4.41]), presence of metastatic solid tumor (OR 2.27 [1.12–4.33]), and decreased pH (OR 0.12 [0.02–0.79]), were also significantly associated with an increased risk for extubation failure (Table 2).

**TABLE 2:** Multivariate logistic regression of risk factors for extubation failure in the train set.

Variables	Estimate	OR (95% CI) per unit	*p*-value
Chronic pulmonary disease (*n*, %)	0.285	1.33 [1.01–1.74]	0.042
Paraplegia (*n*, %)	0.891	2.44 [1.29–4.41]	0.004
Metastatic solid tumor (*n*, %)	0.822	2.27 [1.12–4.33]	0.016
Heart rate (/min)	0.010	1.01 [1.00–1.02]	0.011
Tidal volume (mL)	−0.002	1.00 [1.00–1.00]	<0.001
PEEP (cmH2O)	0.075	1.08 [1.01–1.15]	0.026
Ventilatory ratio	0.749	2.14 [1.65–2.72]	<0.001
pH	−2.083	0.12 [0.02–0.79]	0.028
Blood urea nitrogen (mg/dL)	0.012	1.01 [1.01–1.02]	<0.001
Platelet count (10^9^/L)	0.002	1.00 [1.00–1.00]	0.001
SOFA (score)	0.116	1.12 [1.07–1.17]	<0.001

Definition of abbreviations: OR, odds ratio; CI, confidence interval; PEEP, positive end-expiratory pressure; SOFA, the Sequential Organ Failure Assessment.

**FIGURE 2:**
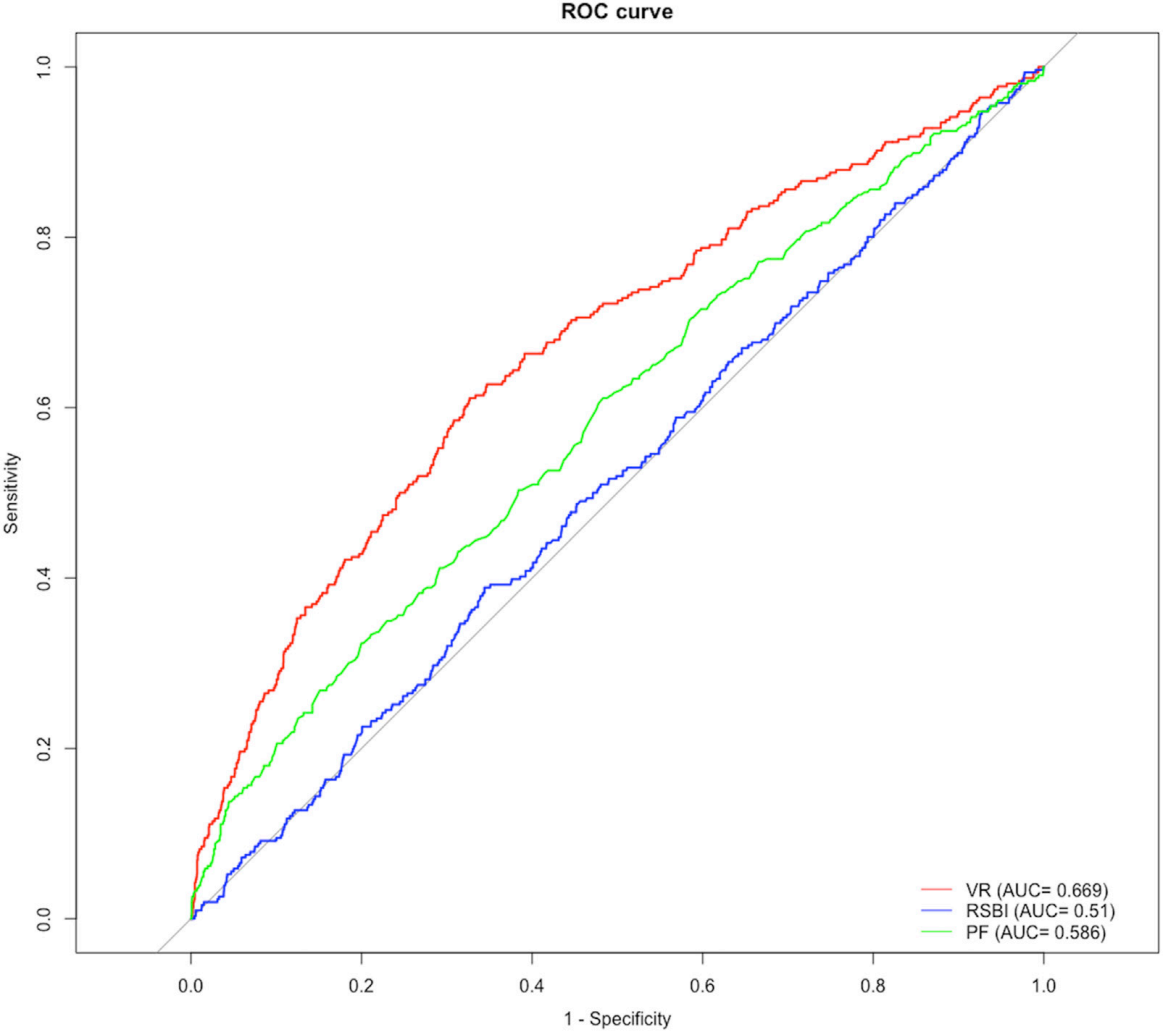
Receiver operating characteristics curve for predictors at 4 h before extubation.

Figure 2 shows ROC curves for VR at 4 hours before extubation (as a continuous variable) in the training set. The area under the ROC curve (AUC) maximized was 0.669 with a cut-off value of 1.595 using the Youden index (Supplementary Table S5). VR ≥ 1.595 remained an independent factor of extubation failure even after adjusting for age, different age groups, type of admission, P_a_O_2_/F_i_O_2_, RSBI, sex, PEEP, and SOFA (Supplementary Table S6), which was confirmed in the validation set (C-index: 0.585 [0.542, 0.628]) and non-surgical group (C-index: 0.632 [0.605–0.658]). Compared with patients with VR < 1.595, patients with VR ≥ 1.595 had prolonged ICU LOS (4 [3, 8] vs. 4 [2, 7] days, *p* = 0.033), a higher risk of extubation failure [78 (19.2%) vs. 70 (10.5%), *p* < 0.001], and higher risk of in-hospital mortality (37 (9.1%) vs. 36 (5.4%), see Table 3). Similar results were found in non-surgical patients (see Supplementary Table S7).

**TABLE 3:** Outcomes of the high-VR group and the low-VR group: data from the validation set.

Variables	Overall	VR < 1.595	VR ≥ 1.595	*p*-value
	1,071	664	407	
ICU LOS (day)	4 [3, 7]	4 [2, 7]	4 [3, 8]	0.033
Hospital LOS (day)	10 [7, 16]	10 [7, 16]	10 [7, 17]	0.425
In-hospital death (*n*, %)	73 (6.8)	36 (5.4)	37 (9.1)	0.029
Extubation failure type (*n*, %)	148 (13.8)	70 (10.5)	78 (19.2)	<0.001
NIV after extubation (*n*, %)	44 (4.1)	22 (3.3)	22 (5.4)	0.13
Reintubation within 48 h (*n*, %)	88 (8.2)	41 (6.2)	47 (11.5)	0.003
Death within 48 h (*n*, %)	26 (2.4)	11 (1.7)	15 (3.7)	0.059

Definition of abbreviations: VR, ventilatory ratio; ICU, intensive care unit; LOS, length of stay; NIV, noninvasive ventilation.

### Secondary outcomes

We also tested the predictive ability of RSBI and P_a_O_2_/F_i_O_2_ in this population, and the AUC of VR was significantly higher than that of RSBI (0.510 [IQR, 0.476–0.545]) and P_a_O_2_/F_i_O_2_ (0.586 [IQR, 0.551–0.621]) at 4 hours before extubation for predicting reintubation (all *p*-value < 0.05, Supplementary Table S5). In addition, significant differences were found between survivors and nonsurvivors in VR (1.44 [1.20, 1.74] vs. 1.49 [1.19, 1.96], *p* = 0.032, see Supplementary Table S8).

## Discussion

The current retrospective study focused on a large cohort of ventilated patients who were admitted to the ICU for at least 48 h. The rate of extubation-failure was 12.7% and the median SOFA score was 6 before extubation. We found that higher VR at 4 hours before extubation was an independent factor for predicting extubation failure even after adjusting for age, sex, type of admission, P_a_O_2_/F_i_O_2_, RSBI, PEEP, and SOFA. The threshold of VR was 1.595 with a predictive value of 0.585 in predicting extubation failure. Other variables, increased heart rate, lowerr tidal volume, greater PEEP levels, decreased pH levels, higher blood urea nitrogen level, elevated platelet count, presence of chronic pulmonary disease, paraplegia, and metastatic solid tumor were also independent factors associated with extubation failure.

We found that a threshold of 1.595 of VR was an independent risk factor for extubation failure in this study. This result was consistent with one previous study that showed that patients were finally liberated from assisted mechanical ventilation only when their VR fell below 2 (16). In addition, we found that the predictability of VR was better than that of RSBI for predicting extubation failure. Although RSBI is commonly used to support decision-making during weaning, it might be significantly affected by the level of ventilator support (Patel et al., 2009). VR was a rather reliable variable even after adjusting variables that increased extubation failure and testing in a non-surgical population. This further supports the ability of VR to identify increased ventilatory demands and the potential to be a better tool to facilitate the decision-making process of extubation.

Several studies reported that VR in the first days of mechanical ventilation was significantly associated with a higher risk of mortality (Sinha et al., 2019; Ende et al., 2021; Torres et al., 2021). In a recent multicenter study involving patients with coronavirus disease 2019 (COVID-19)-related ARDS, it was observed that the VR was significantly different between survivors and non-survivors on the second day (1.95 ± 0.68 vs. 2.09 ± 0.60) and third day (2.06 ± 0.70 vs. 2.26 ± 0.68) of mechanical ventilation (Morales-Quinteros et al., 2021). Moreover, one study showed the dynamic change of VR from ICU admission to day 3 was independently associated with death (OR 1.04 [1.01–1.07], *p* = 0.030) (Torres et al., 2021). In line with those studies, we also found VR 4 hours before extubation was higher in nonsurvivors.

Other factors, such as increased heart rate, evaluated PEEP, higher BUN, higher platelet count, greater SOFA score, decreased pH, decreased tidal volume, and presence of chronic pulmonary disease, paraplegia, and metastatic solid tumor were also significantly associated with an increased risk for extubation failure in the current study. Heart rate and SOFA score were comprehensive factors to evaluate the disease progression of the patient. Patients who required higher PEEP and exhibited a lower tidal volume might be associated with impaired lung compliance and inadequate gas exchange. The levels of BUN and pH reflect the accumulation of waste products in the systemic circulation and the imbalance of the acid-base status. Suraseranivong et al. also reported higher levels of BUN in the group of patients who experienced extubation failure (Suraseranivong et al., 2018). Patients with chronic pulmonary disease, paraplegia, and metastatic solid tumor are often accompanied by respiratory muscle weakness and impaired respiratory functions, including weakened cough reflex and decreased ability to clear secretions. A previous study reported that patients older than 65 years with pre-existing chronic cardiac or respiratory diseases had a significantly higher reintubation rate of 34% compared to 9% in patients without these conditions (Thille et al., 2011).

There are several limitations of this study. First, our study was a retrospective study based on the MIMIC-IV database. The ventilation strategies and settings used before extubation and the protocols of extubation were not standardized. Second, we failed to calculate the duration of ventilation due to the lack of intubation records before extubation. However, we excluded patients after surgery following routine ventilation in sensitivity analysis, as those patients had a shorter ventilation duration, a low risk of extubation failure, and a low incidence of ARDS. Therefore, the incidence of extubation failure in the current study was 12.7% which was similar to that in previous studies, ranging from 10%–20% (Thille et al., 2013). The low fraction of ARDS in the current study might be related to the fact that the diagnosis of ARDS is underestimated in clinical settings. We could not reclassify the included patients according to the Berlin definition since this was a retrospective study, which might lead to biased results. Third, we failed to capture data on levels of sedation, which may have influenced ventilatory impairment. In addition, the acute physiology and chronic health evaluation II score was not calculated because of insufficient data. The amount of missing data in the variables assessed in the study is a potential limitation. However, the analyses after multiple imputations yielded similar results (see Supplementary Table S8). Although our VR cut-off value is validated using data from a multicentre database, we still need prospective studies for further exploration.

## Conclusion

In conclusion, higher VR at 4 hours before extubation was an independent factor for predicting extubation failure within 48 h. VR showed the ability to identify increased ventilatory demands and might be a useful tool to support decision-making during weaning.

## Data Availability

Publicly available datasets were analyzed in this study. This data can be found here: https://mimic.mit.edu/.

## Abbreviations

ABG, arterial blood gas; ARDS: acute respiratory distress syndrome; AUC, area under receiver operating characteristic curve; BMI, body mass index; BUN, blood urea nitrogen; CI, confidence interval; CO2, carbon dioxide; COVID-19, Coronavirus disease 2019; GCS, glasgow coma scales; HFNC, high-flow nasal cannula; ICU, intensive care unit; IMV, invasive mechanical ventilation; IQR, interquartile range; LOS, length of stay; NIV, noninvasive ventilation; MV, mechanical ventilation; OR, odd ratio; PaCO2, the arterial partial pressure of carbon dioxide; PEEP, positive end-expiratory pressure; PaO2/FiO2, the partial pressure of oxygen to the fraction of inspired oxygen; RBC, red blood cell; ROC, receiver-operating characteristic; RSBI: rapid shallow breathing index; SpO2, peripheral arterial oxygen saturation; SOFA, the sequential organ failure assessment; VR, ventilatory ratio.
