# Supplementary material for: Ventilatory ratio as a predictor for extubation failure in critical ill patients based on MIMIC-IV database (from 2008 to 2019)
Source: Front Physiol. 2023 Jun 1;14:1137115. doi: 10.3389/fphys.2023.1137115 (PMC10267390; doi:10.3389/fphys.2023.1137115)

Additional file 1

Ventilatory Ratio as a Predictor for Extubation Failure in Critical Ill Patients based on MIMIC-IV Database (From 2008 to 2019)

Huan, Yang[1#]; Yuenan, Ni[1#]; Dong Huang[1]; Zongan, Liang[1*]

[1] Department of Respiratory and Critical Care Medicine, West China School of Medicine and West China Hospital, Sichuan University, China; [#]Contributed equally.

[*]Corresponding author: Zongan Liang, Department of Respiratory and Critical Care Medicine, West China School of Medicine and West China Hospital, Sichuan University, No. 37 Guoxue Alley, Chengdu, 610041 Sichuan, China. Email: liangza@scu.edu.cn

Table of content

Supplementary Table 1 Baseline population characteristics with stratification by extubation failure before extubation

Supplementary Table 2 Baseline population characteristics with stratification by extubation failure on admission

Supplementary Table 3 Baseline population characteristics between train set and validation set

Supplementary Table 4 Baseline characteristics with stratification by extubation failure before extubation in non-surgical patients

Supplementary Table 5 Predicting power of extubation failure by ventilatory ratio, RSBI, and PaO2/FiO2

Supplementary Table 6 Odds ratio for extubation failure using multivariate logistic regression with ventilatory ratio as the base model in the train set

Supplementary Table 7 Outcomes of the high-VR group and the low-VR group: data from the non-surgical group

Supplementary Table 8 Baseline population characteristics with stratification by in-hospital mortality before extubation.

Supplementary Table 9 Data before and after imputation

Supplementary Table 10 Reasonable ranges for included variables

Supplementary Table 11 Baseline population characteristics with stratification by extubation failure before extubation in train set (full table)

Supplementary Figure 1 Result of lasso regression

Supplementary Table 1 Baseline population characteristics with stratification by extubation failure before extubation

Variables	Overall	Extubation success	Extubation failure	P value
	3569	3115	454	
Age (year, median, IQR)	66.00 [57.00, 76.00]	66.00 [57.00, 76.00]	67.00 [56.00, 76.00]	0.892
Age (year, mean, SD)	65.29 (13.98)	65.27 (13.99)	65.45 (13.87)	0.794
Male (n, %)	2396 (67.1)	2093 (67.2)	303 (66.7)	0.891
BMI (kg/m2)	27.68 [24.16, 31.88]	27.47 [24.14, 31.60]	29.17 [24.38, 34.50]	<0.001
Type of admission (n, %)				<0.001
Emergency	1208 (33.8)	1013 (32.5)	195 (43.0)	
Surgery	699 (19.6)	636 (20.4)	63 (13.9)	
Urgent	916 (25.7)	807 (25.9)	109 (24.0)	
Elective	358 (10.0)	327 (10.5)	31 (6.8)	
Other	388 (10.9)	332 (10.7)	56 (12.3)	
Charlson comorbidity index (score)	6.00 [4.00, 7.00]	6.00 [4.00, 7.00]	6.00 [5.00, 8.00]	<0.001
Age structure				0.842
<50 years	161 (4.5)	142 (4.6)	19 (4.2)	
50-59 years	223 (6.2)	192 (6.2)	31 (6.8)	
60-69 years	573 (16.1)	507 (16.3)	66 (14.5)	
70-79 years	1014 (28.4)	886 (28.4)	128 (28.2)	
⩾80 years	1598 (44.8)	1388 (44.6)	210 (46.3)	
Myocardial infarct (n, %)	995 (27.9)	878 (28.2)	117 (25.8)	0.31
Congestive heart failure (n, %)	1374 (38.5)	1190 (38.2)	184 (40.5)	0.368
Peripheral vascular disease (n, %)	712 (19.9)	626 (20.1)	86 (18.9)	0.609
Cerebrovascular disease (n, %)	429 (12.0)	371 (11.9)	58 (12.8)	0.651
Dementia (n, %)	46 (1.3)	39 (1.3)	7 (1.5)	0.773
Chronic pulmonary disease (n, %)	1014 (28.4)	842 (27.0)	172 (37.9)	<0.001
Rheumatic disease (n, %)	110 (3.1)	99 (3.2)	11 (2.4)	0.469
Peptic ulcer disease (n, %)	82 (2.3)	60 (1.9)	22 (4.8)	<0.001
Paraplegia (n, %)	84 (2.4)	64 (2.1)	20 (4.4)	0.003
Renal disease (n, %)	847 (23.7)	718 (23.0)	129 (28.4)	0.014
Malignant cancer (n, %)	268 (7.5)	227 (7.3)	41 (9.0)	0.222
Metastatic solid tumor (n, %)	81 (2.3)	63 (2.0)	18 (4.0)	0.015
Aids (n, %)	11 (0.3)	10 (0.3)	1 (0.2)	1
Diabetes (n, %)	1236 (34.6)	1067 (34.3)	169 (37.2)	0.234
Liver disease (n, %)	390 (10.9)	318 (10.2)	72 (15.9)	<0.001
ARDS (n, %)	27 (0.8)	19 (0.6)	8 (1.8)	0.018
Heart rate (/min)	88.00 [80.00, 100.00]	88.00 [80.00, 99.00]	93.00 [83.00, 106.00]	<0.001
Mean arterial pressure (mmHg)	85.00 [77.00, 94.00]	85.00 [78.00, 94.00]	85.00 [76.00, 95.88]	0.436
Glasgow coma scale (score)	15.00 [15.00, 15.00]	15.00 [15.00, 15.00]	15.00 [14.25, 15.00]	0.06
SOFA (score)	6.00 [5.00, 9.00]	6.00 [5.00, 8.00]	7.00 [5.00, 10.00]	<0.001
f (/min)	19.00 [16.00, 24.00]	19.00 [16.00, 24.00]	20.00 [17.00, 26.00]	<0.001

Tidal volume (mL)	497.00 [422.00, 587.00]	500.00 [424.00, 591.00]	478.50 [402.25, 557.75]	<0.001
PEEP (cmH2O)	5.00 [5.00, 5.00]	5.00 [5.00, 5.00]	5.00 [5.00, 8.00]	<0.001
Minute volume (L)	8.70 [7.30, 10.60]	8.50 [7.20, 10.30]	9.92 [8.00, 12.28]	<0.001
RSBI (/[min`mL])	41.98 [30.84, 57.78]	41.97 [30.82, 57.74]	42.19 [31.11, 57.79]	0.831
Ventilatory ratio	1.44 [1.20, 1.75]	1.42 [1.19, 1.71]	1.69 [1.34, 2.10]	<0.001
FiO2 (%)	50.00 [40.00, 50.00]	50.00 [40.00, 50.00]	50.00 [40.00, 50.00]	0.15
PaO2 (mmHg)	107.00 [86.00, 130.00]	108.00 [87.00, 131.00]	97.00 [79.00, 121.75]	<0.001
PaO2/FiO2 (mmHg)	244.00 [184.00, 312.50]	247.50 [188.00, 315.00]	220.00 [160.00, 286.88]	<0.001
SpO2 (%)	95.00 [93.00, 97.00]	95.00 [93.00, 97.00]	94.00 [91.00, 96.00]	<0.001
PaCO2 (mmHg)	40.00 [37.00, 45.00]	40.00 [37.00, 45.00]	41.00 [36.00, 48.00]	0.017
A-aDO2 (mmHg)	155.20 [115.95, 215.00]	154.45 [114.95, 213.50]	162.85 [120.53, 220.44]	0.076
pH	7.38 [7.34, 7.42]	7.38 [7.34, 7.42]	7.38 [7.33, 7.42]	0.046
Anion gap (mEq/L)	13.00 [11.00, 16.00]	13.00 [11.00, 16.00]	14.00 [12.00, 18.00]	<0.001
Base excess (mEq/L)	0.00 [-2.00, 1.00]	0.00 [-2.00, 1.00]	0.00 [-2.00, 2.00]	0.842
Blood urea nitrogen (mg/dL)	19.00 [14.00, 30.00]	19.00 [14.00, 28.00]	24.00 [16.00, 42.00]	<0.001
Creatinine (mg/dL)	1.00 [0.80, 1.40]	1.00 [0.80, 1.40]	1.20 [0.80, 2.00]	<0.001
INR	1.40 [1.20, 1.60]	1.40 [1.20, 1.60]	1.40 [1.20, 1.70]	0.781
PT (s)	15.30 [13.70, 17.20]	15.40 [13.80, 17.10]	15.10 [13.30, 18.00]	0.633
Platelet count (109/L)	139.00 [103.00, 188.00]	138.00 [103.00, 185.00]	151.00 [104.00, 216.00]	0.001
Red blood cell count (109/L)	3.55 [3.18, 3.98]	3.57 [3.21, 4.00]	3.43 [3.01, 3.86]	<0.001
White blood cell count (109/L)	14.00 [10.50, 18.90]	14.00 [10.50, 18.80]	14.20 [10.12, 19.87]	0.762
ICU LOS (day)	4.06 [2.76, 7.35]	3.84 [2.53, 6.43]	8.07 [4.18, 15.49]	<0.001
Hospital LOS (day)	9.93 [6.77, 16.05]	9.45 [6.59, 15.02]	13.93 [8.81, 23.21]	<0.001
Extubation failure type (n, %)	454 (12.7)	0 (0.0)	454 (100.0)	<0.001
NIV after extubation (n, %)	141 (4.0)	0 (0.0)	141 (31.1)	<0.001
Death within 48 hours (n, %)	83 (2.3)	0 (0.0)	83 (18.3)	<0.001
Reintubation within 48 hours (n, %)	247 (6.9)	0 (0.0)	247 (54.4)	<0.001
In-hospital death (n, %)	260 (7.3)	129 (4.1)	131 (28.9)	<0.001

Definition of abbreviations: BMI body mass index; ARDS acute respiratory distress syndrome; SOFA the Sequential Organ Failure Assessment; PEEP positive end-expiratory pressure; RSBI rapid shallow breathing index; FiO2 fraction inspired oxygen concentration; PaO2 arterial oxygen partial pressure; SpO2 peripheral arterial oxygen saturation; PaCO2 arterial carbon dioxide partial pressure; A-aDO2 alveolar-arterial oxygen difference; INR international normalized ratio; PT prothrombin time; ICU intensive care unit; LOS length of stay; NIV noninvasive ventilation.

Supplementary Table 2 Baseline population characteristics with stratification by extubation failure on ICU admission

Variables	Overall	Extubation success	Extubation failure	P value
	3569	3115	454	
Age (year)	66.00 [57.00, 76.00]	66.00 [57.00, 76.00]	67.00 [56.00, 76.00]	0.892
Male (n, %)	2396 (67.1)	2093 (67.2)	303 (66.7)	0.891
BMI (kg/m2)	27.68 [24.16, 31.88]	27.47 [24.14, 31.60]	29.17 [24.38, 34.50]	<0.001
Type of admission (n, %)				<0.001
Emergency	1208 (33.8)	1013 (32.5)	195 (43.0)	
Surgery	699 (19.6)	636 (20.4)	63 (13.9)	
Urgent	916 (25.7)	807 (25.9)	109 (24.0)	
Elective	358 (10.0)	327 (10.5)	31 (6.8)	
Other	388 (10.9)	332 (10.7)	56 (12.3)	
Charlson comorbidity index (score)	6.00 [4.00, 7.00]	6.00 [4.00, 7.00]	6.00 [5.00, 8.00]	<0.001
Age structure				0.842
<50 years	161 (4.5)	142 (4.6)	19 (4.2)	
50-59 years	223 (6.2)	192 (6.2)	31 (6.8)	
60-69 years	573 (16.1)	507 (16.3)	66 (14.5)	
70-79 years	1014 (28.4)	886 (28.4)	128 (28.2)	
≥80 years	1598 (44.8)	1388 (44.6)	210 (46.3)	
Myocardial infarct (n, %)	995 (27.9)	878 (28.2)	117 (25.8)	0.31
Congestive heart failure (n, %)	1374 (38.5)	1190 (38.2)	184 (40.5)	0.368
Peripheral vascular disease (n, %)	712 (19.9)	626 (20.1)	86 (18.9)	0.609
Cerebrovascular disease (n, %)	429 (12.0)	371 (11.9)	58 (12.8)	0.651
Dementia (n, %)	46 (1.3)	39 (1.3)	7 (1.5)	0.773
Chronic pulmonary disease (n, %)	1014 (28.4)	842 (27.0)	172 (37.9)	<0.001
Rheumatic disease (n, %)	110 (3.1)	99 (3.2)	11 (2.4)	0.469
Peptic ulcer disease (n, %)	82 (2.3)	60 (1.9)	22 (4.8)	<0.001
Paraplegia (n, %)	84 (2.4)	64 (2.1)	20 (4.4)	0.003
Renal disease (n, %)	847 (23.7)	718 (23.0)	129 (28.4)	0.014
Malignant cancer (n, %)	268 (7.5)	227 (7.3)	41 (9.0)	0.222
Metastatic solid tumor (n, %)	81 (2.3)	63 (2.0)	18 (4.0)	0.015
Aids (n, %)	11 (0.3)	10 (0.3)	1 (0.2)	1
Diabetes (n, %)	1236 (34.6)	1067 (34.3)	169 (37.2)	0.234
Liver disease (n, %)	390 (10.9)	318 (10.2)	72 (15.9)	<0.001
ARDS (n, %)	27 (0.8)	19 (0.6)	8 (1.8)	0.018
Heart rate (/min)	98.00 [88.00, 112.00]	97.00 [88.00, 111.00]	103.00 [89.25, 117.00]	<0.001
Mean arterial pressure (mmHg)	97.00 [88.88, 109.00]	97.00 [88.00, 108.00]	100.00 [90.00, 112.75]	0.007
Glasgow coma scale (score)	15.00 [14.00, 15.00]	15.00 [14.00, 15.00]	15.00 [14.00, 15.00]	0.701
SOFA (score)	6.00 [5.00, 9.00]	6.00 [5.00, 9.00]	7.00 [5.00, 10.00]	<0.001
f (/min)	21.00 [18.00, 26.00]	21.00 [18.00, 25.00]	23.00 [19.50, 27.00]	<0.001
Tidal volume (mL)	550.00 [490.00, 628.00]	551.50 [491.00, 629.00]	534.00 [473.00, 614.00]	0.008
PEEP (cmH2O)	6.00 [5.00, 10.00]	5.70 [5.00, 10.00]	8.00 [5.00, 10.00]	<0.001

Minute volume (L)	9.80 [8.30, 11.80]	9.60 [8.20, 11.40]	12.40 [9.92, 15.00]	<0.001
RSBI (/[min`mL])	45.35 [34.77, 58.96]	45.71 [35.07, 60.40]	42.16 [33.42, 52.11]	<0.001
Ventilatory ratio	1.96 [1.58, 2.47]	1.90 [1.55, 2.35]	2.58 [2.07, 3.34]	<0.001
FiO2 (%)	100.00 [80.00, 100.00]	100.00 [100.00, 100.00]	100.00 [60.00, 100.00]	<0.001
PaO2 (mmHg)	86.00 [65.00, 111.00]	87.00 [68.00, 113.00]	73.00 [48.50, 92.50]	<0.001
PaO2/FiO2 (mmHg)	170.00 [107.50, 246.67]	175.00 [111.00, 252.50]	138.17 [90.00, 202.50]	<0.001
SpO2 (%)	93.00 [91.00, 95.00]	93.00 [91.00, 95.00]	92.00 [90.00, 95.00]	<0.001
PaCO2 (mmHg)	48.00 [43.00, 53.00]	48.00 [43.00, 53.00]	50.00 [44.00, 57.00]	<0.001
A-aDO2 (mmHg)	365.50 [228.76, 495.69]	365.93 [229.50, 493.19]	360.50 [217.00, 507.31]	0.917
pH	7.31 [7.25, 7.35]	7.31 [7.26, 7.35]	7.28 [7.22, 7.34]	<0.001
Anion gap (mEq/L)	14.00 [12.00, 16.00]	14.00 [12.00, 16.00]	15.00 [13.00, 19.00]	<0.001
Base excess (mEq/L)	1.00 [0.00, 3.00]	1.00 [0.00, 3.00]	1.00 [-1.00, 3.00]	0.064
Blood urea nitrogen (mg/dL)	19.00 [15.00, 29.00]	19.00 [14.00, 28.00]	23.50 [17.00, 41.00]	<0.001
Creatinine (mg/dL)	1.10 [0.80, 1.50]	1.10 [0.80, 1.50]	1.20 [0.90, 2.00]	<0.001
INR	1.40 [1.30, 1.60]	1.40 [1.30, 1.60]	1.40 [1.30, 1.80]	0.086
PT (s)	15.70 [14.10, 17.80]	15.60 [14.10, 17.60]	15.90 [13.80, 19.20]	0.218
Platelet count (109/L)	138.00 [103.00, 187.00]	136.00 [102.00, 184.00]	150.50 [107.00, 215.00]	<0.001
Red blood cell count (109/L)	3.63 [3.29, 4.06]	3.63 [3.29, 4.06]	3.64 [3.30, 4.10]	0.754
White blood cell count (109/L)	15.10 [11.20, 19.80]	15.00 [11.20, 19.70]	15.40 [11.30, 20.70]	0.193
ICU LOS (day)	4.06 [2.76, 7.35]	3.84 [2.53, 6.43]	8.07 [4.18, 15.49]	<0.001
Hospital LOS (day)	9.93 [6.77, 16.05]	9.45 [6.59, 15.02]	13.93 [8.81, 23.21]	<0.001
In-hospital death (n, %)	260 (7.3)	129 (4.1)	131 (28.9)	<0.001
Extubation failure type (n, %)	454 (12.7)	0 (0.0)	454 (100.0)	<0.001
NIV after extubation (n, %)	141 (4.0)	0 (0.0)	141 (31.1)	<0.001
Death within 48 hours (n, %)	83 (2.3)	0 (0.0)	83 (18.3)	<0.001
Reintubation within 48 hours (n, %)	247 (6.9)	0 (0.0)	247 (54.4)	<0.001

Definition of abbreviations: BMI body mass index; ARDS acute respiratory distress syndrome; SOFA the Sequential Organ Failure Assessment; PEEP positive end-expiratory pressure; RSBI rapid shallow breathing index; FiO2 fraction inspired oxygen concentration; PaO2 arterial oxygen partial pressure; SpO2 peripheral arterial oxygen saturation; PaCO2 arterial carbon dioxide partial pressure; A-aDO2 alveolar-arterial oxygen difference; INR international normalized ratio; PT prothrombin time; ICU intensive care unit; LOS length of stay; NIV noninvasive ventilation.

Supplementary Table 3 Baseline population characteristics between train set and validation set

Variables	Overall	Train set	Validation set	P value
	3569	2498	1071	
Age (year)	66.00 [57.00, 76.00]	67.00 [57.00, 76.00]	66.00 [57.00, 75.00]	0.682
Male (n, %)	2396 (67.1)	1687 (67.5)	709 (66.2)	0.46
BMI (kg/m2)	27.68 [24.16, 31.88]	27.68 [24.19, 31.88]	27.70 [24.14, 31.98]	0.877
Type of admission (n, %)				1
Emergency	1208 (33.8)	846 (33.9)	362 (33.8)	
Surgery	699 (19.6)	491 (19.7)	208 (19.4)	
Urgent	916 (25.7)	640 (25.6)	276 (25.8)	
Elective	358 (10.0)	251 (10.0)	107 (10.0)	
Other	388 (10.9)	270 (10.8)	118 (11.0)	
Charlson comorbidity index (score)	6.00 [4.00, 7.00]	6.00 [4.00, 8.00]	6.00 [4.00, 7.00]	0.552
Age structure				0.33
<50 years	161 (4.5)	117 (4.7)	44 (4.1)	
50-59 years	223 (6.2)	151 (6.0)	72 (6.7)	
60-69 years	573 (16.1)	411 (16.5)	162 (15.1)	
70-79 years	1014 (28.4)	688 (27.5)	326 (30.4)	
≥80 years	1598 (44.8)	1131 (45.3)	467 (43.6)	
Myocardial infarct (n, %)	995 (27.9)	688 (27.5)	307 (28.7)	0.519
Congestive heart failure (n, %)	1374 (38.5)	968 (38.8)	406 (37.9)	0.662
Peripheral vascular disease (n, %)	712 (19.9)	507 (20.3)	205 (19.1)	0.456
Cerebrovascular disease (n, %)	429 (12.0)	297 (11.9)	132 (12.3)	0.756
Dementia (n, %)	46 (1.3)	37 (1.5)	9 (0.8)	0.163
Chronic pulmonary disease (n, %)	1014 (28.4)	712 (28.5)	302 (28.2)	0.885
Rheumatic disease (n, %)	110 (3.1)	82 (3.3)	28 (2.6)	0.341
Peptic ulcer disease (n, %)	82 (2.3)	52 (2.1)	30 (2.8)	0.233
Paraplegia (n, %)	84 (2.4)	67 (2.7)	17 (1.6)	0.063
Renal disease (n, %)	847 (23.7)	602 (24.1)	245 (22.9)	0.457
Malignant cancer (n, %)	268 (7.5)	187 (7.5)	81 (7.6)	0.991
Metastatic solid tumor (n, %)	81 (2.3)	59 (2.4)	22 (2.1)	0.658
Aids (n, %)	11 (0.3)	6 (0.2)	5 (0.5)	0.429
Diabetes (n, %)	1236 (34.6)	858 (34.3)	378 (35.3)	0.613
Liver disease (n, %)	390 (10.9)	274 (11.0)	116 (10.8)	0.95
ARDS (n, %)	27 (0.8)	24 (1.0)	3 (0.3)	0.052
Heart rate (/min)	88.00 [80.00, 100.00]	88.00 [80.00, 100.00]	88.00 [80.00, 100.00]	0.614
Mean arterial pressure (mmHg)	85.00 [77.00, 94.00]	85.00 [77.00, 94.38]	86.00 [78.00, 94.00]	0.511
Glasgow coma scale (score)	15.00 [15.00, 15.00]	15.00 [15.00, 15.00]	15.00 [15.00, 15.00]	0.812
SOFA (score)	6.00 [5.00, 9.00]	6.00 [5.00, 9.00]	6.00 [5.00, 9.00]	0.574
f (/min)	19.00 [16.00, 24.00]	19.00 [16.00, 24.00]	19.00 [16.00, 23.00]	0.346
Tidal volume (mL)	497.00 [422.00, 587.00]	496.00 [421.00, 588.75]	498.00 [422.00, 586.00]	0.888

PEEP (cmH2O)	5.00 [5.00, 5.00]	5.00 [5.00, 5.00]	5.00 [5.00, 5.00]	0.561
Minute volume (L)	8.70 [7.30, 10.60]	8.70 [7.30, 10.60]	8.60 [7.20, 10.50]	0.437
RSBI (/[min`mL])	41.98 [30.84, 57.78]	42.02 [30.83, 57.63]	41.72 [30.84, 58.01]	0.742
Ventilatory ratio	1.44 [1.20, 1.75]	1.44 [1.21, 1.75]	1.44 [1.18, 1.76]	0.773
FiO2 (%)	50.00 [40.00, 50.00]	50.00 [40.00, 50.00]	50.00 [40.00, 50.00]	0.77
PaO2 (mmHg)	107.00 [86.00, 130.00]	107.00 [86.00, 131.00]	106.00 [86.50, 129.00]	0.595
PaO2/FiO2 (mmHg)	244.00 [184.00, 312.50]	244.00 [184.00, 314.00]	245.00 [187.50, 310.00]	0.879
SpO2 (%)	95.00 [93.00, 97.00]	95.00 [93.00, 97.00]	95.00 [93.00, 97.00]	0.862
PaCO2 (mmHg)	40.00 [37.00, 45.00]	40.00 [36.00, 45.00]	40.00 [37.00, 45.00]	0.334
A-aDO2 (mmHg)	155.20 [115.95, 215.00]	155.70 [115.70, 216.44]	154.20 [117.10, 210.75]	0.583
pH	7.38 [7.34, 7.42]	7.38 [7.34, 7.42]	7.38 [7.34, 7.42]	0.561
Anion gap (mEq/L)	13.00 [11.00, 16.00]	13.00 [11.00, 16.00]	13.00 [11.00, 16.00]	0.708
Base excess (mEq/L)	0.00 [-2.00, 1.00]	0.00 [-2.00, 1.00]	0.00 [-2.00, 1.00]	0.674
Blood urea nitrogen (mg/dL)	19.00 [14.00, 30.00]	19.00 [14.00, 30.00]	19.00 [14.00, 30.00]	0.927
Creatinine (mg/dL)	1.00 [0.80, 1.40]	1.00 [0.80, 1.50]	1.00 [0.80, 1.40]	0.618
INR	1.40 [1.20, 1.60]	1.40 [1.20, 1.60]	1.40 [1.20, 1.60]	0.18
PT (s)	15.30 [13.70, 17.20]	15.30 [13.70, 17.10]	15.40 [13.80, 17.30]	0.369
Platelet count (109/L)	139.00 [103.00, 188.00]	138.00 [102.00, 186.00]	141.00 [105.00, 192.50]	0.071
Red blood cell count (109/L)	3.55 [3.18, 3.98]	3.55 [3.16, 3.99]	3.56 [3.22, 3.95]	0.753
White blood cell count (109/L)	14.00 [10.50, 18.90]	14.10 [10.50, 18.90]	13.90 [10.50, 18.90]	0.927
ICU LOS (day)	4.06 [2.76, 7.35]	4.10 [2.81, 7.32]	3.96 [2.55, 7.46]	0.491
Hospital LOS (day)	9.93 [6.77, 16.05]	9.90 [6.81, 16.01]	9.98 [6.60, 16.13]	0.953
In-hospital death (n, %)	260 (7.3)	187 (7.5)	73 (6.8)	0.525
Extubation failure type (n, %)	454 (12.7)	306 (12.2)	148 (13.8)	0.217
NIV after extubation (n, %)	141 (4.0)	97 (3.9)	44 (4.1)	0.824
Death within 48 hours (n, %)	83 (2.3)	57 (2.3)	26 (2.4)	0.886
Reintubation within 48 hours (n, %)	247 (6.9)	159 (6.4)	88 (8.2)	0.054

Definition of abbreviations: BMI body mass index; ARDS acute respiratory distress syndrome; SOFA the Sequential Organ Failure Assessment; PEEP positive end-expiratory pressure; RSBI rapid shallow breathing index; FiO2 fraction inspired oxygen concentration; PaO2 arterial oxygen partial pressure; SpO2 peripheral arterial oxygen saturation; PaCO2 arterial carbon dioxide partial pressure; A-aDO2 alveolar-arterial oxygen difference; INR international normalized ratio; PT prothrombin time; ICU intensive care unit; LOS length of stay; NIV noninvasive ventilation.

Supplementary Table 4 Baseline characteristics with stratification by extubation failure before extubation in non-surgical patients

Variables	Overall<br>2870	Extubation success<br>2479	Extubation failure<br>391	P value
Age (year)	66.00 [56.00, 76.00]	66.00 [56.00, 76.00]	67.00 [56.00, 76.00]	0.452
Male (n, %)	1903 (66.3)	1647 (66.4)	256 (65.5)	0.751
BMI (kg/m2)	27.43 [24.01, 31.80]	27.30 [24.00, 31.48]	28.87 [24.07, 34.31]	0.001
Type of admission (n, %)				0.001
Emergency	1208 (42.1)	1013 (40.9)	195 (49.9)	
Urgent	916 (31.9)	807 (32.6)	109 (27.9)	
Elective	358 (12.5)	327 (13.2)	31 (7.9)	
Other	388 (13.5)	332 (13.4)	56 (14.3)	
Charlson comorbidity index (score)	6.00 [4.00, 8.00]	6.00 [4.00, 8.00]	6.00 [5.00, 8.00]	<0.001
Age structure				0.654
<50 years	145 (5.1)	128 (5.2)	17 (4.3)	
50-59 years	189 (6.6)	162 (6.5)	27 (6.9)	
60-69 years	470 (16.4)	415 (16.7)	55 (14.1)	
70-79 years	772 (26.9)	663 (26.7)	109 (27.9)	
⩾80 years	1294 (45.1)	1111 (44.8)	183 (46.8)	
Myocardial infarct (n, %)	859 (29.9)	753 (30.4)	106 (27.1)	0.211
Congestive heart failure (n, %)	1203 (41.9)	1033 (41.7)	170 (43.5)	0.536
Peripheral vascular disease (n, %)	547 (19.1)	472 (19.0)	75 (19.2)	1
Cerebrovascular disease (n, %)	359 (12.5)	307 (12.4)	52 (13.3)	0.67
Dementia (n, %)	41 (1.4)	34 (1.4)	7 (1.8)	0.675
Chronic pulmonary disease (n, %)	825 (28.7)	683 (27.6)	142 (36.3)	<0.001
Rheumatic disease (n, %)	83 (2.9)	75 (3.0)	8 (2.0)	0.362
Peptic ulcer disease (n, %)	79 (2.8)	57 (2.3)	22 (5.6)	<0.001
Paraplegia (n, %)	78 (2.7)	59 (2.4)	19 (4.9)	0.008
Renal disease (n, %)	710 (24.7)	598 (24.1)	112 (28.6)	0.062
Malignant cancer (n, %)	215 (7.5)	179 (7.2)	36 (9.2)	0.199
Metastatic solid tumor (n, %)	64 (2.2)	48 (1.9)	16 (4.1)	0.012
Aids (n, %)	11 (0.4)	10 (0.4)	1 (0.3)	1
Diabetes (n, %)	1008 (35.1)	859 (34.7)	149 (38.1)	0.203
Liver disease (n, %)	359 (12.5)	291 (11.7)	68 (17.4)	0.002
ARDS (n, %)	25 (0.9)	18 (0.7)	7 (1.8)	0.07
Heart rate (/min)	88.00 [80.00, 101.00]	88.00 [80.00, 100.00]	93.00 [83.50, 106.50]	<0.001
Mean aterial pressure (mmHg)	85.00 [77.00, 95.00]	85.50 [78.00, 95.00]	85.00 [75.00, 98.25]	0.433
Glasgow coma scale (score)	15.00 [15.00, 15.00]	15.00 [15.00, 15.00]	15.00 [14.00, 15.00]	0.039
SOFA (score)	7.00 [5.00, 9.00]	7.00 [5.00, 9.00]	8.00 [6.00, 10.00]	<0.001
f (/min)	19.00 [16.00, 24.00]	19.00 [16.00, 24.00]	21.00 [17.00, 26.00]	<0.001
Tidal volume (mL)	493.00 [418.00, 585.00]	497.00 [420.00, 589.00]	474.00 [400.00, 552.50]	<0.001

PEEP (cmH2O)	5.00 [5.00, 5.00]	5.00 [5.00, 5.00]	5.00 [5.00, 7.50]	<0.001
Minute volume (L)	8.70 [7.30, 10.60]	8.50 [7.20, 10.30]	9.94 [7.97, 12.50]	<0.001
RSBI (/[min`mL])	42.45 [31.08, 57.85]	42.11 [30.84, 57.62]	43.88 [31.62, 60.10]	0.35
Ventilatory ratio	1.44 [1.19, 1.75]	1.41 [1.18, 1.70]	1.69 [1.34, 2.09]	<0.001
FiO2 (%)	50.00 [40.00, 50.00]	50.00 [40.00, 50.00]	50.00 [40.00, 50.00]	0.15
PaO2 (mmHg)	106.00 [86.00, 129.00]	107.00 [87.00, 130.00]	97.00 [78.00, 123.00]	<0.001
PaO2/FiO2 (mmHg)	245.00 [184.00, 312.50]	247.50 [188.00, 315.50]	222.00 [159.00, 291.25]	<0.001
SpO2 (%)	95.00 [93.00, 97.00]	95.00 [93.00, 97.00]	94.00 [91.00, 96.00]	<0.001
PaCO2 (mmHg)	40.00 [36.00, 45.00]	40.00 [36.00, 45.00]	40.00 [36.00, 48.00]	0.127
A-aDO2 (mmHg)	152.45 [114.20, 212.00]	152.00 [113.45, 210.75]	154.75 [118.12, 219.50]	0.128
pH	7.39 [7.35, 7.42]	7.39 [7.35, 7.42]	7.38 [7.33, 7.43]	0.02
Anion gap (mEq/L)	14.00 [11.00, 16.00]	13.00 [11.00, 16.00]	15.00 [12.00, 18.50]	<0.001
Base excess (mEq/L)	0.00 [-2.00, 1.00]	0.00 [-2.00, 1.00]	0.00 [-3.00, 2.00]	0.572
Blood urea nitrogen (mg/dL)	20.00 [15.00, 32.00]	20.00 [15.00, 30.00]	26.00 [17.00, 46.00]	<0.001
Creatinine (mg/dL)	1.10 [0.80, 1.50]	1.00 [0.80, 1.50]	1.30 [0.90, 2.10]	<0.001
INR	1.40 [1.20, 1.60]	1.40 [1.20, 1.60]	1.40 [1.20, 1.70]	0.604
PT (s)	15.40 [13.70, 17.40]	15.40 [13.80, 17.25]	15.20 [13.40, 18.70]	0.754
Platelet count (109/L)	139.50 [102.00, 193.00]	138.00 [102.00, 189.00]	152.00 [101.50, 216.00]	0.008
Red blood cell count (109/L)	3.54 [3.14, 4.00]	3.56 [3.17, 4.03]	3.39 [2.96, 3.83]	<0.001
White blood cell count (109/L)	13.80 [10.20, 18.80]	13.80 [10.20, 18.70]	14.10 [9.90, 20.05]	0.662
ICU LOS (day)	4.29 [2.97, 8.18]	4.10 [2.85, 7.06]	8.65 [4.27, 15.86]	<0.001
Hospital LOS (day)	11.03 [7.55, 17.47]	10.72 [7.28, 16.53]	14.51 [9.09, 23.64]	<0.001
In-hospital death (n, %)	250 (8.7)	120 (4.8)	130 (33.2)	<0.001
Extubation failure type (n, %)	391 (13.6)	0 (0.0)	391 (100.0)	<0.001
NIV after extubation (n, %)	110 (3.8)	0 (0.0)	110 (28.1)	<0.001
Death within 48 hours (n, %)	83 (2.9)	0 (0.0)	83 (21.2)	<0.001
Reintubation within 48 hours (n, %)	212 (7.4)	0 (0.0)	212 (54.2)	<0.001

Definition of abbreviations: BMI body mass index; ARDS acute respiratory distress syndrome; SOFA the Sequential Organ Failure Assessment; PEEP positive end-expiratory pressure; RSBI rapid shallow breathing index; FiO2 fraction inspired oxygen concentration; PaO2 arterial oxygen partial pressure; SpO2 peripheral arterial oxygen saturation; PaCO2 arterial carbon dioxide partial pressure; A-aDO2 alveolar-arterial oxygen difference; INR international normalized ratio; PT prothrombin time; ICU intensive care unit; LOS length of stay; NIV noninvasive ventilation.

Supplementary Table 5 Predicting power of extubation failure by ventilatory ratio, RSBI, and PaO2/FiO2

	Train set					P	Validation set	Non-surgical group
	Cutoff value	AUC (95% CI)	Youden Index	Specificity	Sensitivity		AUC (95% CI)	AUC (95% CI)
VR	1.595	0.669 [0.635-0.703]	0.284	0.673	0.611	<0.001*	0.585 [0.542-0.628]	0.632 [0.605-0.658]
RSBI (breaths/min/L)	50.69	0.510 [0.476-0.545]	0.045	0.656	0.389	<0.001**	0.487 [0.447-0.528]	0.516 [0.490-0.541]
PaO2/FiO2 (mmHg)	243.25	0.586 [0.551-0.621]	0.128	0.517	0.611	0.002#	0.556 [0.514-0.599]	0.553 [0.527-0.580]

Definition of abbreviations: AUC area under receiver operating characteristic curve; CI confidence interval; VR ventilatory ratio; RSBI rapid shallow beath index; P_aO_2/F_iO_2 the partial pressure of oxygen to the fraction of inspired oxygen.

Notes: * VR vs. RSBI; ** VR vs. PaO2/FiO2; # RSBI vs. PaO2/FiO2

Supplementary Table 6 Odds ratio for extubation failure using multivariate logistic regression with ventilatory ratio as the base model in the train set

	OR (95% CI)	P
Univariate analysis		
Ventilatory ratio≥1.595 (base model)	3.37 [2.63-4.34]	<0.001
Multivariate analysis		
Base model+ age	3.24 [2.53-4.15]	<0.001
Base model+ age structure	3.25 [2.54-4.17]	<0.001
Base model+ PaO2/FiO2	3.03 [2.36-3.90]	<0.001
Base model+ RSBI	3.33 [2.60-4.29]	<0.001
Base model+ sex	3.31 [2.59-4.25]	<0.001
Base model+ type of admission	3.20 [2.50-4.11]	<0.001
Base model+ PEEP	3.01 [2.34-3.87]	<0.001
Base model+ SOFA	3.37 [2.63-4.34]	<0.001

Notes: This Table only shows ORs of ventilatory ratio in each model.

Definition of abbreviations: OR odds ratio; CI confidence interval; P_aO_2/F_iO_2 the partial pressure of oxygen to the fraction of inspired oxygen; RSBI rapid shallow beath index; SOFA the Sequential Organ Failure Assessment; PEEP positive end-expiratory pressure.

Supplementary Table 7 Outcomes of the high-VR group and the low-VR group: data from the non-surgical group

Variables	Overall	VR<1.595	VR≥1.595	P value
	2870	1834	1036	
ICU LOS (day)	4.29 [2.97, 8.18]	4.15 [2.87, 7.71]	4.86 [3.10, 9.39]	<0.001
Hospital LOS (day)	11.03 [7.55, 17.47]	10.85 [7.31, 17.07]	11.80 [7.75, 17.79]	0.107
In-hospital death (n, %)	250 (8.7)	144 (7.9)	106 (10.2)	0.035
Extubation failure type (n, %)	391 (13.6)	161 (8.8)	230 (22.2)	<0.001
NIV after extubation (n, %)	110 (3.8)	47 (2.6)	63 (6.1)	<0.001
Reintubation within 48 hours (n, %)	212 (7.4)	87 (4.7)	125 (12.1)	<0.001
Death within 48 hours (n, %)	83 (2.9)	31 (1.7)	52 (5.0)	<0.001

Definition of abbreviations: VR ventilatory ratio; ICU intensive care unit; LOS length of stay; NIV noninvasive ventilation.

Supplementary Table 8 Baseline population characteristics with stratification by in-hospital mortality before extubation.

Variables	Overall	Survivors	Non-survivors	P value
	3569	3309	260	
Age (year)	66.00 [57.00, 76.00]	66.00 [57.00, 75.00]	69.50 [58.00, 78.25]	0.008
Male (n, %)	2396 (67.1)	2237 (67.6)	159 (61.2)	0.039
BMI (kg/m2)	27.68 [24.16, 31.88]	27.76 [24.30, 32.00]	26.17 [22.56, 30.96]	<0.001
Type of admission (n, %)				<0.001
Emergency	1208 (33.8)	1074 (32.5)	134 (51.5)	
Surgery	699 (19.6)	689 (20.8)	10 (3.8)	
Urgent	916 (25.7)	848 (25.6)	68 (26.2)	
Elective	358 (10.0)	351 (10.6)	7 (2.7)	
Other	388 (10.9)	347 (10.5)	41 (15.8)	
Charlson comorbidity index (score)	6.00 [4.00, 7.00]	6.00 [4.00, 7.00]	7.00 [5.00, 9.00]	<0.001
Age structure				0.022
<50 years	161 (4.5)	149 (4.5)	12 (4.6)	
50-59 years	223 (6.2)	207 (6.3)	16 (6.2)	
60-69 years	573 (16.1)	538 (16.3)	35 (13.5)	
70-79 years	1014 (28.4)	958 (29.0)	56 (21.5)	
⩾80 years	1598 (44.8)	1457 (44.0)	141 (54.2)	
Myocardial infarct (n, %)	995 (27.9)	917 (27.7)	78 (30.0)	0.471
Congestive heart failure (n, %)	1374 (38.5)	1254 (37.9)	120 (46.2)	0.01
Peripheral vascular disease (n, %)	712 (19.9)	644 (19.5)	68 (26.2)	0.012
Cerebrovascular disease (n, %)	429 (12.0)	395 (11.9)	34 (13.1)	0.656
Dementia (n, %)	46 (1.3)	38 (1.1)	8 (3.1)	0.018
Chronic pulmonary disease (n, %)	1014 (28.4)	934 (28.2)	80 (30.8)	0.421
Rheumatic disease (n, %)	110 (3.1)	98 (3.0)	12 (4.6)	0.194
Peptic ulcer disease (n, %)	82 (2.3)	57 (1.7)	25 (9.6)	<0.001
Paraplegia (n, %)	84 (2.4)	75 (2.3)	9 (3.5)	0.312
Renal disease (n, %)	847 (23.7)	761 (23.0)	86 (33.1)	<0.001
Malignant cancer (n, %)	268 (7.5)	231 (7.0)	37 (14.2)	<0.001
Metastatic solid tumor (n, %)	81 (2.3)	61 (1.8)	20 (7.7)	<0.001
Aids (n, %)	11 (0.3)	9 (0.3)	2 (0.8)	0.417
Diabetes (n, %)	1236 (34.6)	1154 (34.9)	82 (31.5)	0.307
Liver disease (n, %)	390 (10.9)	310 (9.4)	80 (30.8)	<0.001
ARDS (n, %)	27 (0.8)	19 (0.6)	8 (3.1)	<0.001
Heart rate (/min)	88.00 [80.00, 100.00]	88.00 [80.00, 99.00]	92.00 [81.00, 109.00]	<0.001
Mean arterial pressure (mmHg)	85.00 [77.00, 94.00]	85.00 [78.00, 94.00]	81.00 [73.00, 96.25]	<0.001
Glasgow coma scale (score)	15.00 [15.00, 15.00]	15.00 [15.00, 15.00]	15.00 [14.00, 15.00]	0.011
SOFA (score)	6.00 [5.00, 9.00]	6.00 [5.00, 8.00]	9.50 [7.00, 13.00]	<0.001
f (/min)	19.00 [16.00, 24.00]	19.00 [16.00, 24.00]	21.00 [17.00, 26.00]	0.001
Tidal volume (mL)	497.00 [422.00, 587.00]	500.00 [425.00, 590.00]	463.00 [382.75, 548.50]	<0.001

PEEP (cmH2O)	5.00 [5.00, 5.00]	5.00 [5.00, 5.00]	5.00 [5.00, 8.00]	<0.001
Minute volume (L)	8.70 [7.30, 10.60]	8.70 [7.30, 10.50]	9.11 [7.38, 11.53]	0.021
RSBI (/[min`mL])	41.98 [30.84, 57.78]	41.56 [30.62, 57.30]	46.45 [33.31, 63.49]	0.003
Ventilatory ratio	1.44 [1.20, 1.75]	1.44 [1.20, 1.74]	1.49 [1.19, 1.96]	0.032
FiO2 (%)	50.00 [40.00, 50.00]	50.00 [40.00, 50.00]	40.00 [40.00, 50.00]	<0.001
PaO2 (mmHg)	107.00 [86.00, 130.00]	107.00 [87.00, 131.00]	102.50 [78.00, 125.25]	0.008
PaO2/FiO2 (mmHg)	244.00 [184.00, 312.50]	244.00 [186.00, 312.00]	242.50 [156.19, 317.86]	0.14
SpO2 (%)	95.00 [93.00, 97.00]	95.00 [93.00, 97.00]	94.00 [91.00, 96.25]	<0.001
PaCO2 (mmHg)	40.00 [37.00, 45.00]	40.00 [37.00, 45.00]	40.00 [35.00, 48.00]	0.325
A-aDO2 (mmHg)	155.20 [115.95, 215.00]	155.70 [116.95, 215.00]	146.45 [100.26, 216.50]	0.092
pH	7.38 [7.34, 7.42]	7.38 [7.34, 7.42]	7.37 [7.30, 7.42]	0.001
Anion gap (mEq/L)	13.00 [11.00, 16.00]	13.00 [11.00, 15.00]	16.00 [13.00, 21.00]	<0.001
Base excess (mEq/L)	0.00 [-2.00, 1.00]	0.00 [-2.00, 1.00]	-1.00 [-5.00, 1.00]	<0.001
Blood urea nitrogen (mg/dL)	19.00 [14.00, 30.00]	19.00 [14.00, 28.00]	32.00 [18.00, 50.25]	<0.001
Creatinine (mg/dL)	1.00 [0.80, 1.40]	1.00 [0.80, 1.40]	1.60 [0.90, 2.70]	<0.001
INR	1.40 [1.20, 1.60]	1.40 [1.20, 1.60]	1.50 [1.30, 2.02]	<0.001
PT (s)	15.30 [13.70, 17.20]	15.30 [13.70, 17.10]	16.45 [13.97, 22.25]	<0.001
Platelet count (109/L)	139.00 [103.00, 188.00]	139.00 [104.00, 188.00]	128.00 [69.50, 194.00]	0.001
Red blood cell count (109/L)	3.55 [3.18, 3.98]	3.58 [3.21, 4.00]	3.21 [2.82, 3.62]	<0.001
White blood cell count (109/L)	14.00 [10.50, 18.90]	14.00 [10.50, 18.80]	14.95 [10.20, 20.90]	0.08
ICU LOS (day)	4.06 [2.76, 7.35]	3.96 [2.62, 6.85]	8.90 [4.45, 15.63]	<0.001
Hospital LOS (day)	9.93 [6.77, 16.05]	9.84 [6.77, 15.75]	11.87 [6.77, 21.35]	0.01
In-hospital death (n, %)	260 (7.3)	0 (0.0)	260 (100.0)	<0.001
Extubation failure type (n, %)	454 (12.7)	323 (9.8)	131 (50.4)	<0.001
NIV after extubation (n, %)	141 (4.0)	133 (4.0)	8 (3.1)	0.558
Death within 48 hours (n, %)	83 (2.3)	0 (0.0)	83 (31.9)	<0.001
Reintubation within 48 hours (n, %)	247 (6.9)	200 (6.0)	47 (18.1)	<0.001

Definition of abbreviations: BMI body mass index; ARDS acute respiratory distress syndrome; SOFA the Sequential Organ Failure Assessment; PEEP positive end-expiratory pressure; RSBI rapid shallow breathing index; FiO2 fraction inspired oxygen concentration; PaO2 arterial oxygen partial pressure; SpO2 peripheral arterial oxygen saturation; PaCO2 arterial carbon dioxide partial pressure; A-aDO2 alveolar-arterial oxygen difference; INR international normalized ratio; PT prothrombin time; ICU intensive care unit; LOS length of stay; NIV noninvasive ventilation.

Supplementary Table 9 Data before and after imputation

Variables	Overall	Before imputation	After imputation	P value
	5740	2870	2870	
Age (year)	66.00 [56.00, 76.00]	66.00 [56.00, 76.00]	66.00 [56.00, 76.00]	1
Male (n, %)	3806 (66.3)	1903 (66.3)	1903 (66.3)	1
BMI (kg/m2)	27.44 [24.01, 31.80]	27.44 [24.02, 31.79]	27.43 [24.00, 31.80]	0.978
Type of admission (n, %)				1
Emergency	2416 (42.1)	1208 (42.1)	1208 (42.1)	
Surgery	1832 (31.9)	916 (31.9)	916 (31.9)	
Urgent	716 (12.5)	358 (12.5)	358 (12.5)	
Elective	776 (13.5)	388 (13.5)	388 (13.5)	
Other				1
Charlson comorbidity index (score)	6.00 [4.00, 8.00]	6.00 [4.00, 8.00]	6.00 [4.00, 8.00]	1
Age structure	290 (5.1)	145 (5.1)	145 (5.1)	
<50 years	378 (6.6)	189 (6.6)	189 (6.6)	
50-59 years	940 (16.4)	470 (16.4)	470 (16.4)	
60-69 years	1544 (26.9)	772 (26.9)	772 (26.9)	
70-79 years	2588 (45.1)	1294 (45.1)	1294 (45.1)	
⩾80 years	1718 (29.9)	859 (29.9)	859 (29.9)	1
Myocardial infarct (n, %)	2406 (41.9)	1203 (41.9)	1203 (41.9)	1
Congestive heart failure (n, %)	1094 (19.1)	547 (19.1)	547 (19.1)	1
Peripheral vascular disease (n, %)	718 (12.5)	359 (12.5)	359 (12.5)	1
Cerebrovascular disease (n, %)	82 (1.4)	41 (1.4)	41 (1.4)	1
Dementia (n, %)	1650 (28.7)	825 (28.7)	825 (28.7)	1
Chronic pulmonary disease (n, %)	166 (2.9)	83 (2.9)	83 (2.9)	1
Rheumatic disease (n, %)	158 (2.8)	79 (2.8)	79 (2.8)	1
Peptic ulcer disease (n, %)	678 (11.8)	339 (11.8)	339 (11.8)	1
Paraplegia (n, %)	1420 (24.7)	710 (24.7)	710 (24.7)	1
Renal disease (n, %)	430 (7.5)	215 (7.5)	215 (7.5)	1
Malignant cancer (n, %)	310 (5.4)	155 (5.4)	155 (5.4)	1
Metastatic solid tumor (n, %)	22 (0.4)	11 (0.4)	11 (0.4)	1
Aids (n, %)	2016 (35.1)	1008 (35.1)	1008 (35.1)	1
Diabetes (n, %)	718 (12.5)	359 (12.5)	359 (12.5)	1
Liver disease (n, %)	50 (0.9)	25 (0.9)	25 (0.9)	1
ARDS (n, %)				1
Heart rate (/min)	88.00 [80.00, 101.00]	88.00 [80.00, 101.00]	88.00 [80.00, 101.00]	1
Mean arterial pressure (mmHg)	85.00 [77.00, 95.00]	85.00 [77.00, 95.00]	85.00 [77.00, 95.00]	1
Glasgow coma scale (score)	15.00 [15.00, 15.00]	15.00 [15.00, 15.00]	15.00 [15.00, 15.00]	0.982
SOFA (score)	7.00 [5.00, 9.00]	7.00 [5.00, 9.00]	7.00 [5.00, 9.00]	1
f (/min)	19.00 [16.00, 24.00]	20.00 [16.00, 24.00]	19.00 [16.00, 24.00]	0.958
Tidal volume (mL)	493.00 [418.00, 584.75]	493.00 [418.00, 584.00]	493.00 [418.00, 585.00]	0.95

PEEP (cmH2O)	5.00 [5.00, 5.00]	5.00 [5.00, 5.00]	5.00 [5.00, 5.00]	0.857
Minute volume (L)	8.70 [7.30, 10.60]	8.70 [7.30, 10.60]	8.70 [7.30, 10.60]	1
RSBI (/[min`mL])	42.45 [31.08, 57.86]	42.45 [31.08, 57.85]	42.45 [31.08, 57.85]	1
Ventilatory ratio	1.44 [1.19, 1.75]	1.44 [1.19, 1.75]	1.44 [1.19, 1.75]	1
FiO2 (%)	50.00 [40.00, 50.00]	50.00 [40.00, 50.00]	50.00 [40.00, 50.00]	0.98
PaO2 (mmHg)	106.00 [86.00, 129.00]	106.00 [86.00, 129.00]	106.00 [86.00, 129.00]	0.985
PaO2/FiO2 (mmHg)	245.00 [184.00, 312.50]	245.00 [184.00, 312.50]	245.00 [184.00, 312.50]	0.869
SpO2 (%)	95.00 [93.00, 97.00]	95.00 [93.00, 97.00]	95.00 [93.00, 97.00]	0.979
PaCO2 (mmHg)	40.00 [36.00, 45.00]	40.00 [36.00, 45.00]	40.00 [36.00, 45.00]	0.986
A-aDO2 (mmHg)	152.70 [114.20, 212.00]	152.95 [114.33, 212.12]	152.20 [113.78, 211.75]	0.773
pH	7.39 [7.35, 7.42]	7.39 [7.35, 7.42]	7.39 [7.35, 7.42]	0.984
Anion gap (mEq/L)	14.00 [11.00, 16.00]	14.00 [11.00, 16.00]	14.00 [11.00, 16.00]	0.735
Base excess (mEq/L)	0.00 [-2.00, 1.00]	0.00 [-2.00, 1.00]	0.00 [-2.00, 1.00]	0.981
Blood urea nitrogen (mg/dL)	20.00 [15.00, 32.00]	20.00 [15.00, 32.00]	20.00 [15.00, 32.00]	0.984
Creatinine (mg/dL)	1.10 [0.80, 1.50]	1.10 [0.80, 1.50]	1.10 [0.80, 1.50]	0.982
INR	1.40 [1.20, 1.60]	1.40 [1.20, 1.60]	1.40 [1.20, 1.60]	0.079
PT (s)	15.40 [13.70, 17.40]	15.40 [13.80, 17.40]	15.30 [13.60, 17.30]	0.092
Platelet count (109/L)	139.00 [102.00, 193.00]	139.00 [102.00, 193.00]	139.00 [102.00, 193.00]	0.985
Red blood cell count (109/L)	3.54 [3.14, 4.00]	3.54 [3.14, 4.00]	3.54 [3.14, 4.00]	0.974
White blood cell count (109/L)	13.80 [10.20, 18.80]	13.80 [10.20, 18.80]	13.80 [10.20, 18.80]	0.99
ICU LOS (day)	4.29 [2.97, 8.18]	4.29 [2.97, 8.18]	4.29 [2.97, 8.18]	1
Hospital LOS (day)	11.03 [7.55, 17.48]	11.03 [7.55, 17.47]	11.03 [7.55, 17.47]	1
In-hospital death (n, %)				
Extubation failure type (n, %)	362 (6.3)	181 (6.3)	181 (6.3)	1
NIV after extubation (n, %)	166 (2.9)	83 (2.9)	83 (2.9)	1
Death within 48 hours (n, %)	424 (7.4)	212 (7.4)	212 (7.4)	1
Reintubation within 48 hours (n, %)	782 (13.6)	391 (13.6)	391 (13.6)	1

Definition of abbreviations: BMI body mass index; ARDS acute respiratory distress syndrome; SOFA the Sequential Organ Failure Assessment; PEEP positive end-expiratory pressure; RSBI rapid shallow breathing index; FiO2 fraction inspired oxygen concentration; PaO2 arterial oxygen partial pressure; SpO2 peripheral arterial oxygen saturation; PaCO2 arterial carbon dioxide partial pressure; A-aDO2 alveolar-arterial oxygen difference; INR international normalized ratio; PT prothrombin time; ICU intensive care unit; LOS length of stay; NIV noninvasive ventilation.

Supplementary Table 10 Reasonable ranges for included variables

Variables	Unit	Normal ranges	Reasonable ranges
Age	years	—	—
Gender		—	—
Weight	kg	—	20-500
Height	cm	—	50-250
BMI	kg/m2	—	—
Admission type	—	—	—
Charlson comorbidity index	score	0-37	0-37
Myocardial infarct	—	—	—
Congestive heart failure	—	—	—
Peripheral vascular disease	—	—	—
Cerebrovascular disease	—	—	—
Chronic pulmonary disease	—	—	—
Peptic ulcer disease	—	—	—
Paraplegia	—	—	—
Renal disease	—	—	—
Malignant cancer	—	—	—
Metastatic solid tumor	—	—	—
AIDS	—	—	—
Diabetes	—	—	—
Liver disease	—	—	—
ARDS	—	—	—
Heart rate	beats/min	—	0-300
Mean arterial pressure	mmHg	—	0-300
Glasgow coma scale	score	3-15	3-15
SOFA score	score	—	0-24
Tidal volume	mL		50-4000
Minute volume	L/min	—	0-200
Respiratory rate total/f	times/min	—	5-70
PEEP	cmH2O	—	0-200
pH	—	7.35-7.45	6-8
FiO2	%	—	21-100
PCO2	mmHg	35-45	0-200
SpO2	%	—	0-100
PaO2	mmHg	85-105	0-1000
PaO2/FiO2	mmHg	—	>0
Base excess	mEq/L	-2-2	—
A-aDO2	mmHg	—	—
Anion gap	mEq/L	8~20	-50-100

Blood urea nitrogen	mg/dL	6~20	0-300
Creatinine	mg/dL	0.4-1.1	0-150
INR	—	0.9-1.1	0-50
PT	sec	9.4-12.5	0-150
Platelet count	109/L	150-400	0-10000
White blood cell count	109/L	4~10	0-1000
Red blood cell count	109/L	—	0-1000

Definition of abbreviations: BMI body mass index; ARDS acute respiratory distress syndrome; SOFA the Sequential Organ Failure Assessment; PEEP positive end-expiratory pressure; RSBI rapid shallow breathing index; FiO2 fraction inspired oxygen concentration; PaO2 arterial oxygen partial pressure; SpO2 peripheral arterial oxygen saturation; PaCO2 arterial carbon dioxide partial pressure; A-aDO2 alveolar-arterial oxygen difference; INR international normalized ratio; PT prothrombin time.

Supplementary Table 11 Baseline population characteristics with stratification by extubation failure before extubation in train set (full table)

Variables	Overall	Extubation success	Extubation failure	P value
	2498	2192	306	
Age (year)	67.00 [57.00, 76.00]	67.00 [57.00, 76.00]	66.00 [56.00, 74.00]	0.414
Male (n, %)	1687 (67.5)	1476 (67.3)	211 (69.0)	0.616
BMI (kg/m2)	27.68 [24.19, 31.88]	27.47 [24.13, 31.56]	29.17 [24.80, 34.40]	<0.001
Type of admission (n, %)				<0.001
Emergency	846 (33.9)	714 (32.6)	132 (43.1)	
Surgery	491 (19.7)	445 (20.3)	46 (15.0)	
Urgent	640 (25.6)	571 (26.0)	69 (22.5)	
Elective	251 (10.0)	232 (10.6)	19 (6.2)	
Other	270 (10.8)	230 (10.5)	40 (13.1)	
Charlson comorbidity index (score)	6.00 [4.00, 8.00]	6.00 [4.00, 7.00]	6.00 [5.00, 8.00]	<0.001
Age structure				0.963
<50 years	117 (4.7)	103 (4.7)	14 (4.6)	
50-59 years	151 (6.0)	132 (6.0)	19 (6.2)	
60-69 years	411 (16.5)	364 (16.6)	47 (15.4)	
70-79 years	688 (27.5)	599 (27.3)	89 (29.1)	
⩾80 years	1131 (45.3)	994 (45.3)	137 (44.8)	
Myocardial infarct (n, %)	688 (27.5)	613 (28.0)	75 (24.5)	0.23
Congestive heart failure (n, %)	968 (38.8)	851 (38.8)	117 (38.2)	0.893
Peripheral vascular disease (n, %)	507 (20.3)	449 (20.5)	58 (19.0)	0.584
Cerebrovascular disease (n, %)	297 (11.9)	262 (12.0)	35 (11.4)	0.868
Dementia (n, %)	37 (1.5)	33 (1.5)	4 (1.3)	0.987
Chronic pulmonary disease (n, %)	712 (28.5)	598 (27.3)	114 (37.3)	<0.001
Rheumatic disease (n, %)	82 (3.3)	74 (3.4)	8 (2.6)	0.597
Peptic ulcer disease (n, %)	52 (2.1)	41 (1.9)	11 (3.6)	0.077
Paraplegia (n, %)	67 (2.7)	50 (2.3)	17 (5.6)	0.002
Renal disease (n, %)	602 (24.1)	515 (23.5)	87 (28.4)	0.069
Malignant cancer (n, %)	187 (7.5)	156 (7.1)	31 (10.1)	0.078
Metastatic solid tumor (n, %)	59 (2.4)	44 (2.0)	15 (4.9)	0.003
Aids (n, %)	6 (0.2)	6 (0.3)	0 (0.0)	0.77
Diabetes (n, %)	858 (34.3)	737 (33.6)	121 (39.5)	0.048
Liver disease (n, %)	274 (11.0)	223 (10.2)	51 (16.7)	0.001
ARDS (n, %)	24 (1.0)	17 (0.8)	7 (2.3)	0.026
Heart rate (/min)	88.00 [80.00, 100.00]	88.00 [80.00, 99.00]	92.50 [84.00, 104.75]	<0.001
Mean arterial pressure (mmHg)	85.00 [77.00, 94.38]	85.00 [77.00, 94.00]	85.00 [76.00, 95.88]	0.61
Glasgow coma scale (score)	15.00 [15.00, 15.00]	15.00 [15.00, 15.00]	15.00 [14.00, 15.00]	0.047
SOFA (score)	6.00 [5.00, 9.00]	6.00 [5.00, 8.00]	7.50 [5.00, 10.00]	<0.001
f (/min)	19.00 [16.00, 24.00]	19.00 [16.00, 24.00]	21.00 [17.00, 26.75]	<0.001
Tidal volume (mL)	496.00 [421.00, 588.75]	500.00 [424.00, 594.00]	479.50 [400.25, 556.75]	0.002

PEEP (cmH2O)	5.00 [5.00, 5.00]	5.00 [5.00, 5.00]	5.00 [5.00, 8.00]	<0.001
Minute volume (L)	8.70 [7.30, 10.60]	8.60 [7.30, 10.30]	10.17 [8.20, 12.30]	<0.001
RSBI (/[min`mL])	42.02 [30.83, 57.63]	41.97 [30.82, 57.60]	43.49 [31.11, 58.70]	0.564
Ventilatory ratio (median, IQR)	1.44 [1.21, 1.75]	1.41 [1.19, 1.71]	1.71 [1.36, 2.10]	<0.001
Ventilatory ratio (mean, min-max)	1.53 (0.53-12.20)	1.49 (0.53-12.20)	1.83 (0.65-4.81)	
FiO2 (%)	50.00 [40.00, 50.00]	50.00 [40.00, 50.00]	50.00 [40.00, 50.00]	0.135
PaO2 (mmHg)	107.00 [86.00, 131.00]	108.00 [87.00, 132.00]	97.00 [79.25, 121.75]	<0.001
PaO2/FiO2 (mmHg)	244.00 [184.00, 314.00]	247.50 [187.50, 317.50]	222.00 [159.25, 284.38]	<0.001
SpO2 (%)	95.00 [93.00, 97.00]	95.00 [93.00, 97.00]	94.00 [92.00, 96.00]	<0.001
PaCO2 (mmHg)	40.00 [36.00, 45.00]	40.00 [36.00, 45.00]	42.00 [36.00, 48.00]	0.006
A-aDO2 (mmHg)	155.70 [115.70, 216.44]	154.72 [114.85, 215.00]	163.22 [121.45, 227.06]	0.07
pH	7.38 [7.34, 7.42]	7.38 [7.34, 7.42]	7.37 [7.33, 7.42]	0.005
Anion gap (mEq/L)	13.00 [11.00, 16.00]	13.00 [11.00, 16.00]	14.00 [12.00, 18.00]	<0.001
Base excess (mEq/L)	0.00 [-2.00, 1.00]	0.00 [-2.00, 1.00]	0.00 [-3.00, 2.00]	0.281
Blood urea nitrogen (mg/dL)	19.00 [14.00, 30.00]	19.00 [14.00, 28.00]	23.00 [16.00, 41.00]	<0.001
Creatinine (mg/dL)	1.00 [0.80, 1.50]	1.00 [0.80, 1.40]	1.20 [0.80, 2.00]	<0.001
INR	1.40 [1.20, 1.60]	1.40 [1.20, 1.60]	1.40 [1.20, 1.70]	0.94
PT (s)	15.30 [13.70, 17.10]	15.30 [13.80, 17.00]	15.10 [13.40, 18.08]	0.908
Platelet count (109/L)	138.00 [102.00, 186.00]	137.00 [102.00, 184.00]	150.50 [98.50, 216.75]	0.017
Red blood cell count (109/L)	3.55 [3.16, 3.99]	3.56 [3.18, 4.00]	3.44 [3.00, 3.91]	0.002
White blood cell count (109/L)	14.10 [10.50, 18.90]	14.10 [10.50, 18.80]	14.10 [10.10, 20.08]	0.98
ICU LOS (day)	4 [3, 7]	4 [3, 7]	8 [4, 15]	<0.001
Hospital LOS (day)	10 [7, 16]	10 [7, 15]	14 [9, 23]	<0.001
In-hospital death (n, %)	187 (7.5)	96 (4.4)	91 (29.7)	<0.001
Extubation failure type (n, %)	306 (12.2)	0 (0.0)	306 (100.0)	<0.001
NIV after extubation (n, %)	97 (3.9)	0 (0.0)	97 (31.7)	<0.001
Death within 48 hours (n, %)	57 (2.3)	0 (0.0)	57 (18.6)	<0.001
Reintubation within 48 hours (n, %)	159 (6.4)	0 (0.0)	159 (52.0)	<0.001

Definition of abbreviations: BMI body mass index; ARDS acute respiratory distress syndrome; SOFA the Sequential Organ Failure Assessment; PEEP positive end-expiratory pressure; RSBI rapid shallow breathing index; FiO2 fraction inspired oxygen concentration; PaO2 arterial oxygen partial pressure; SpO2 peripheral arterial oxygen saturation; PaCO2 arterial carbon dioxide partial pressure; A-aDO2 alveolar-arterial oxygen difference; INR international normalized ratio; PT prothrombin time; ICU intensive care unit; LOS length of stay; NIV noninvasive ventilation.

Supplementary Figure 1 Result of lasso regression

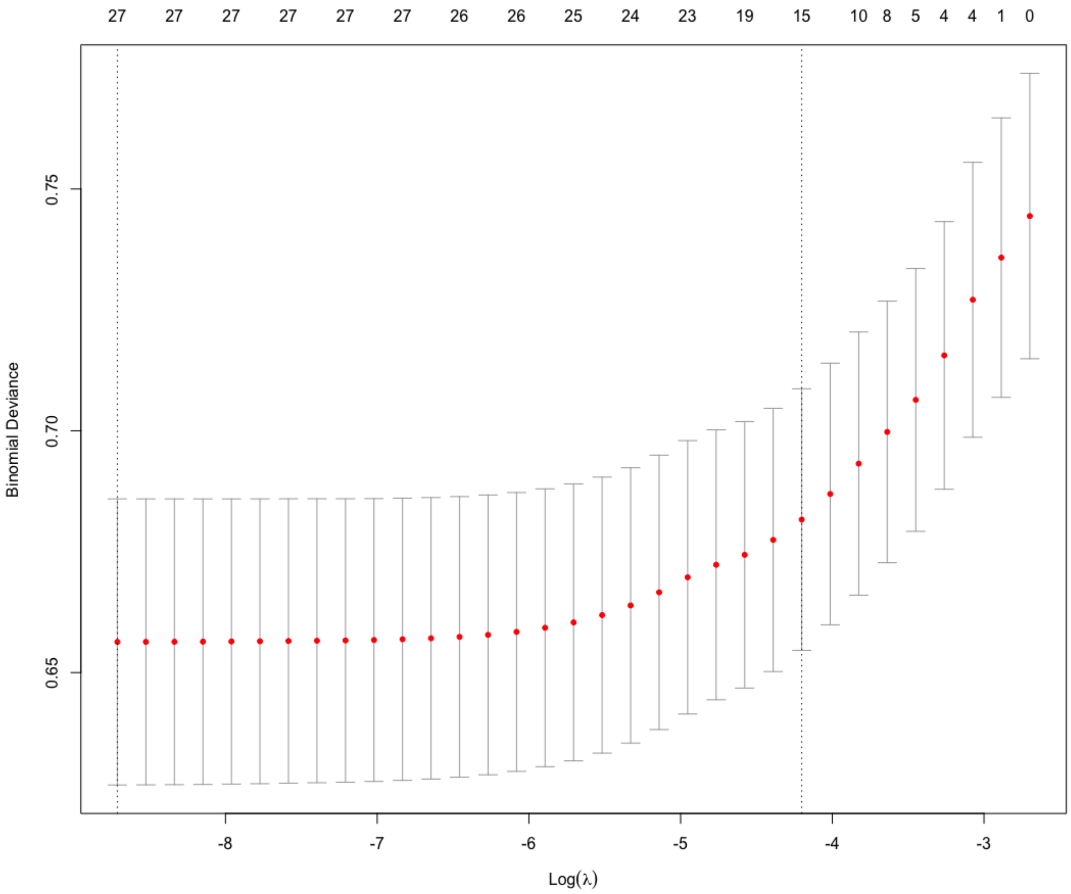

Supplement: Supplementary file 1 [file DataSheet1.pdf]
